# Large-Scale Protein Analysis of Experimental Retinal Artery Occlusion

**DOI:** 10.3390/ijms24097919

**Published:** 2023-04-27

**Authors:** Nanna Vestergaard, Lasse Jørgensen Cehofski, Alexander Nørgård Alsing, Anders Kruse, Jonas Ellegaard Nielsen, Anders Schlosser, Grith Lykke Sorensen, Bent Honoré, Henrik Vorum

**Affiliations:** 1 Department of Ophthalmology, Aalborg University Hospital, 9000 Aalborg, Denmark; noergaard.alsing@gmail.com (A.N.A.); anders.kruse@rn.dk (A.K.); henrik.vorum@rn.dk (H.V.); 2 Department of Clinical Medicine, Aalborg University, 9000 Aalborg, Denmark; bh@biomed.au.dk; 3 Department of Ophthalmology, Odense University Hospital, 5000 Odense, Denmark; lassecehofski@hotmail.com; 4 Department of Clinical Research, University of Southern Denmark, 5000 Odense, Denmark; 5 Department of Clinical Biochemistry, Aalborg University Hospital, 9000 Aalborg, Denmark; j.ellegaard@rn.dk; 6 Department of Cancer and Inflammation Research, Institute of Molecular Medicine, University of Southern Denmark, 5000 Odense C, Denmark; aschlosser@health.sdu.dk (A.S.); glsorensen@health.sdu.dk (G.L.S.); 7 Department of Biomedicine, Aarhus University, 8000 Aarhus, Denmark

**Keywords:** retinal artery occlusion, proteomics, mass spectrometry, animal models, retinal ischemia diseases

## Abstract

Retinal artery occlusion (RAO) is a devastating condition with no effective treatment. The management of RAO could potentially be improved through an in-depth understanding of the molecular alterations in the condition. This study combined advanced proteomic techniques and an experimental model to uncover the retinal large-scale protein profile of RAO. In 13 pigs, RAO was induced with an argon laser and confirmed by fluorescein angiography. Left eyes serving as controls received a sham laser without inducing occlusion. Retinal samples were collected after one, three, or six days and analyzed with liquid chromatography—tandem mass spectrometry. In RAO, 36 proteins were differentially regulated on day one, 86 on day three, and 557 on day six. Upregulated proteins included clusterin, vitronectin, and vimentin, with several proteins increasing over time with a maximum on day six, including clusterin, vimentin, osteopontin, annexin-A, signal transducer, and the activator of transcription 3. On day six, RAO resulted in the upregulation of proteins involved in cellular response to stress, hemostasis, innate immune response, and cytokine signaling. Downregulated proteins were involved in transmission across chemical synapses and visual phototransduction. This study identified the upregulation of multiple inflammatory proteins in RAO and the downregulation of proteins involved in visual pathways.

## 1. Introduction

Retinal artery occlusion (RAO) is a devastating ophthalmic emergency. The majority of cases are associated with severe and irreversible vision loss resulting from the infarction of the inner retinal layers [1]. Although various treatment strategies for acute management have been studied, no treatment has yet been shown to be effective [2,3].

It is a huge challenge to explore therapies for retinal diseases [4]. Recently, new ocular nanotherapeutics with multiple bioactive properties for potential use in the management of retinal disorders have been developed [5]. A basic prerequisite for the development of effective treatment modalities is to know the molecular mechanisms underlying retinal cellular damage. However, these mechanisms are not yet fully understood. Although few previous studies on animal models of RAO have been performed with the identification of certain molecules that are suggested to play a role, large-scale protein analyses of RAO have not yet been performed, and to the best of our knowledge, no method for such analyses have been established [6,7,8,9,10]. Proteomic techniques provide an in-depth analysis of involved proteins in the condition under study and has previously been applied in other ocular diseases to bring new insights into pathological mechanisms and potential therapeutic targets that can be used in clinical practice [6].

To conduct experimental animal studies on RAO in general, a variety of different methodological approaches have previously been used [11]. To increase the translational value of animal models, it is crucial to imitate human disease as closely as possible. Hence, occlusion of the arteries may be performed in pigs by a laser without adjuvants, as this was found to be a suitable model of RAO in a recent review [11]. Therefore, this method was selected in this study to develop and implement an animal model of RAO that was applicable for large-scale protein analyses and thereby generated new insight into the pathological processes occurring in RAO.

Here, we report for the first time on retinal large-scale protein changes in RAO, analyzing an experimental model of laser-induced RAO with advanced proteomic techniques. RAO was induced in a porcine model by applying a laser directly onto a branch retinal artery. The retinas were then dissected, and samples were prepared for liquid chromatography—tandem mass spectrometry (LC-MS/MS). These results were validated using immunohistochemistry.

## 2. Results

### 2.1. Induction of Occlusion

**Figure 1:**
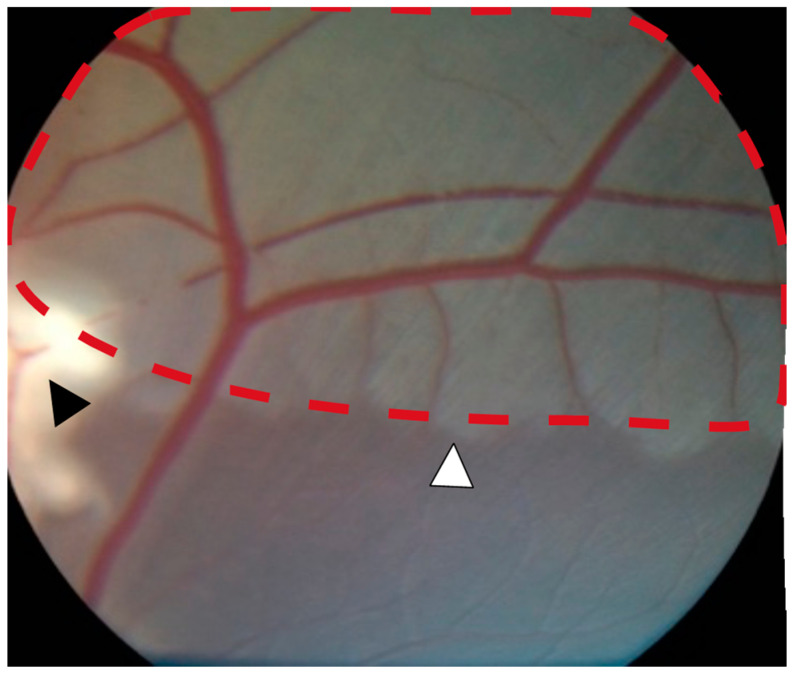
Severe retinal ischemia and ischemic edema (red dotted line) were observed downstream of the occlusion site (black arrowhead). A well-defined watershed zone between ischemic and non-ischemic retina was observed (white arrowhead).

**Figure 2:**
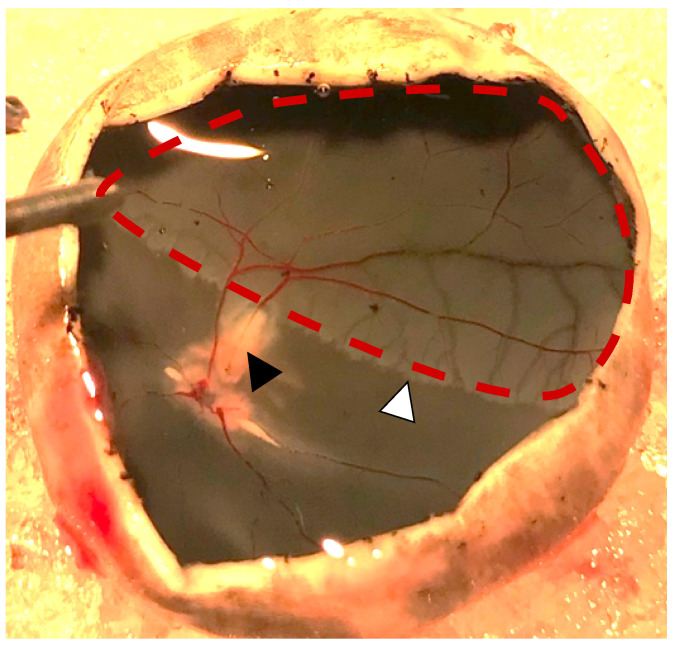
Photo obtained one day after induced retinal artery occlusion (black arrowhead). Severe retinal ischemia is seen downstream of the occlusion (red dotted line). A watershed zone can be observed between the ischemic retina and retinal tissue with normal perfusion (white arrowhead).

**Figure 3:**
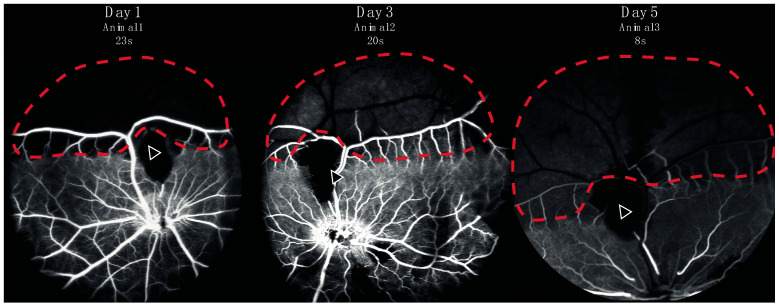
Fluorescein angiography from three representative animals performed in eyes with retinal artery occlusion one, three, or five days after induction of the occlusion. Retinal capillary non-perfusion (red dotted line) was observed downstream of the site of occlusion (arrow). A watershed zone between can be seen at the border between retinal non-perfusion and normal perfusion.

Successful occlusion was induced in the right eyes of all pigs (Figure 1 and Figure 2). Retinal ischemia with ischemic edema was observed in areas supplied by the occluded artery. Manifest “box-carring” and the segmentation of blood flow were observed downstream of the occlusion (Figure 1). Fluorescein angiography (FA) showed the impaired filling of the occluded artery (Figure 3). The FA of all pigs are shown in Supplementary Figure S1. Severe retinal non-perfusion was observed downstream of the occlusion, consistent with retinal ischemia, and was successfully induced in RAO (Figure 3). No reperfusion was seen until day five, when some reperfusion and/or retrograde filling began to appear. A representative FA and fundus photograph of the control eyes are presented in Supplementary Figure S2.

### 2.2. Retinal Protein Expression at Various Days after RAO

**Figure 4:**
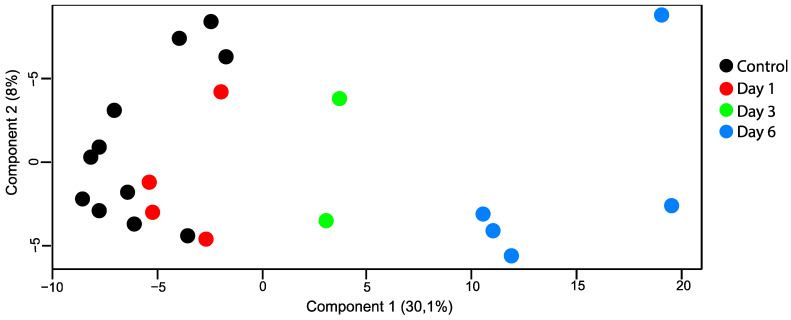
PCA plot (principal component analysis). Retinal samples with retinal artery occlusion collected at day three and day six could be distinguished from the control samples based on their proteomes. There was little overlap between samples with retinal artery occlusion on day one and control samples.

After filtering, a total of 3189 proteins were identified by LC-MS/MS, and 2360 proteins were identified and quantified in at least 70% of the samples within each group (Tables S1 and S2). The data output from MaxQuant is available in Table S3. The principal component analysis (PCA) plot showed how day one tended to produce marginal separation from the controls, whereas day three was clearly distinguished from the control retinas, and day six was even more so (Figure 4).

**Figure 5:**
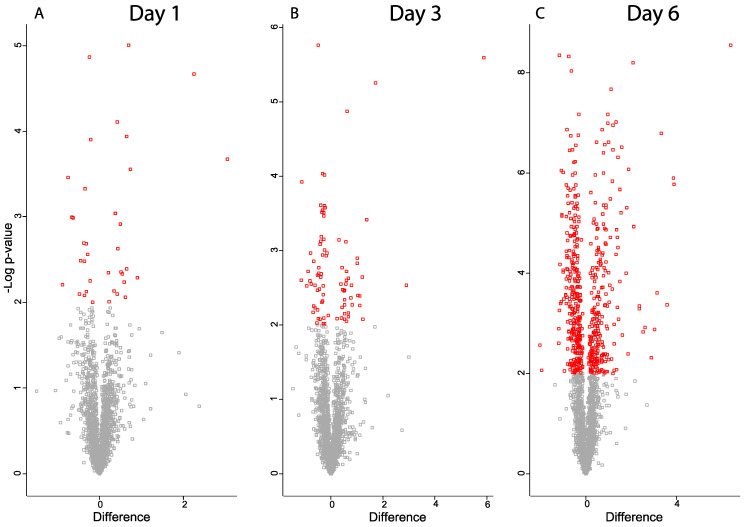
Volcano plots for proteins from ischemic retina samples compared to controls (**A**) day one, (**B**) day three, (**C**) day six. Significantly regulated proteins with *p*-value < 0.05 are colored in red. *X*-axis corresponds to log_2_(Fold-change).

The number of proteins with a statistically significant change between the control and ischemic retina groups was 36 on day one, 86 on day three, and 557 on day six (Figure 5, Tables S4–S6). The protein changes on day six included 251 upregulated and 306 downregulated proteins. On day six, 74 proteins were upregulated with a fold change > 1.75 (Table 1).

**Figure 6:**
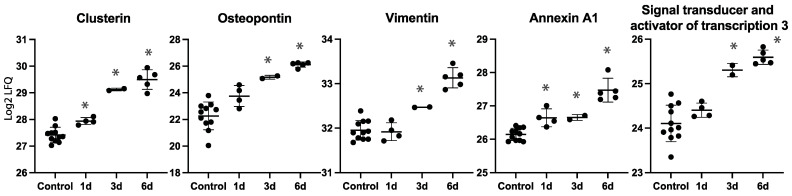
Label-free quantification (LFQ) intensities for selected proteins on each time point. * Denotes statistically significant upregulated proteins compared to controls.

Several of the regulated proteins on day six were also identified as being regulated on day one or three (Table 1). A number of proteins, including clusterin, osteopontin, annexin A1, and the signal transducer and activator of transcription 3 (STAT3), showed increasing label-free quantification (LFQ) values over time with a maximum observed intensity on day six (Figure 6).

### 2.3. Protein–Protein Interaction Network

**Figure 7:**
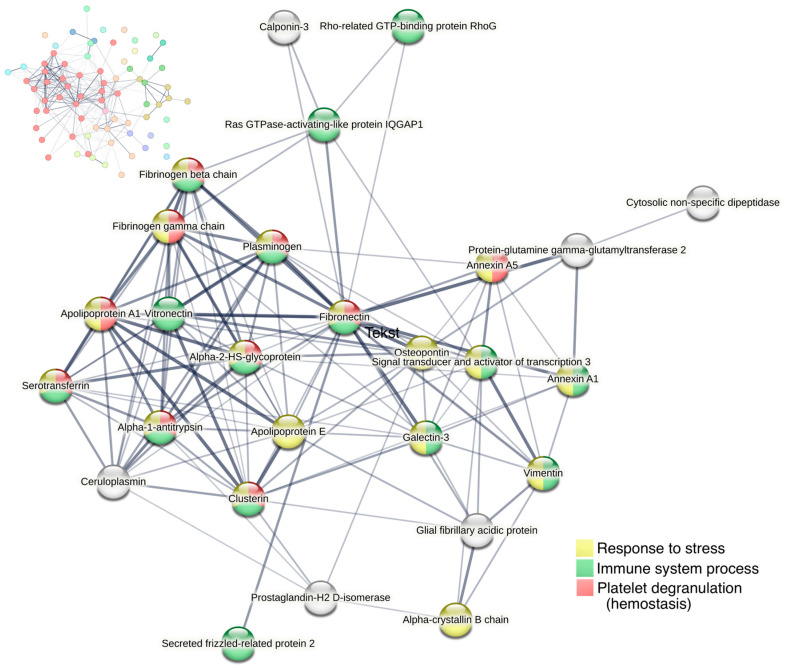
Cluster analysis of protein–protein interactions for proteins with fold change > 1.75 on day six (74 proteins) using STRING. All proteins with >1.75-fold upregulation are shown in the upper left corner, with the largest cluster displayed in red nodes. The largest cluster, displayed in the center of the figure, consists of 27 proteins. Line thickness of the edges indicates the strength of data support. Nodes are colored according to biological processes (Gene Ontology).

A protein–protein interaction network was created for the most upregulated proteins on day six, and more specifically for 74 proteins with a fold-change > 1.75 using STRING. Among these proteins, a major cluster of 27 proteins was identified, including vitronectin, vimentin, glial fibrillary acidic protein (GFAP), clusterin, galectin-3, STAT3, annexin-A1, and osteopontin (Figure 7). The large majority were involved in either the immune system process, platelet degranulation, and/or response to stress.

### 2.4. Functional Enrichment Analyses

To gain further insights into the potential functional implications of differentially expressed proteins, functional enrichment analyses were performed on the up- and downregulated proteins on day six. The number of statistically significant enriched Reactome pathways with a false discovery rate (FDR) < 0.05 were 234 for the upregulated proteins and 166 for the downregulated proteins (Tables S7 and S8). Proteins that were not found in Reactome are listed in Table S9.

**Figure 8:**
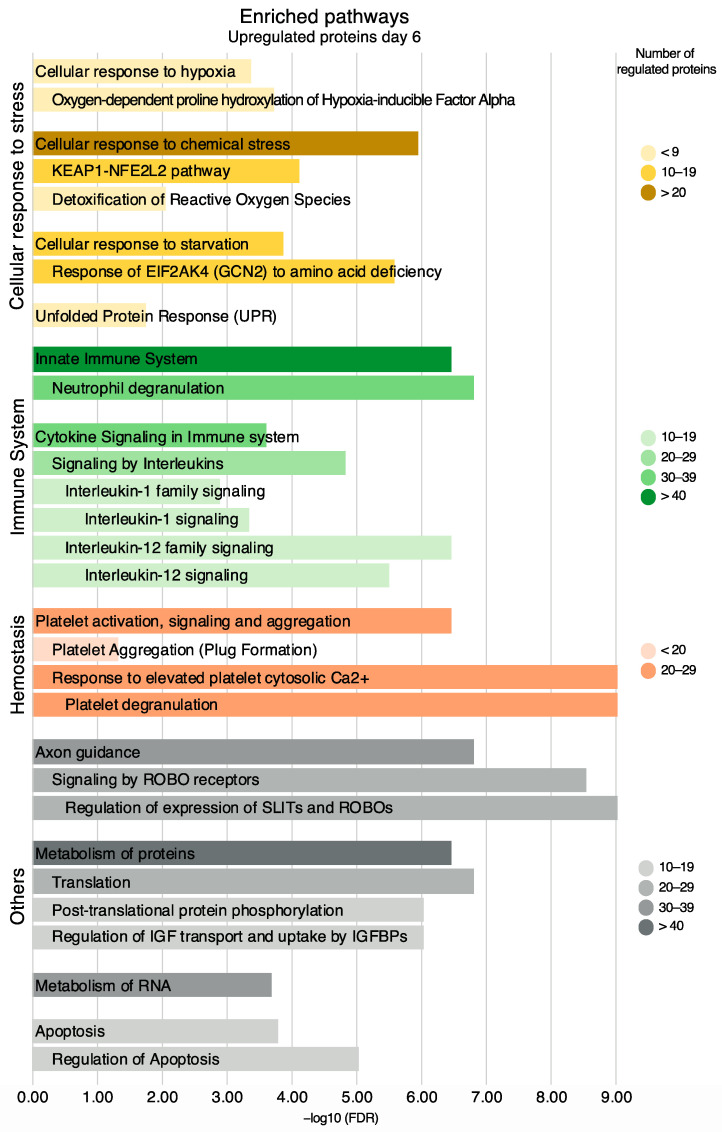
Enriched pathways and their sub-pathways based on all upregulated proteins on day six, non-exhaustive. Pathways are presented in accordance with the Reactome hierarchical organization. Coloring refers to the number of regulated proteins, and the *x*-axis is the statistical significance expressed as −log_10_(FDR).

Among the upregulated proteins, enriched pathways included the immune system (82 proteins, FDR 6.48 × 10^−6^), cellular response to stress (49 proteins, FDR 1.92 × 10^−9^), and hemostasis (29 proteins, FDR 6.77 × 10^−3^) which are presented along with the most significant sub-pathways in a hierarchical manner (Figure 8). Other upregulated pathways included axon guidance, the metabolism of proteins, the metabolism of RNA, and apoptosis (Figure 8). All proteins in these pathways are listed in Table S10.

**Figure 9:**
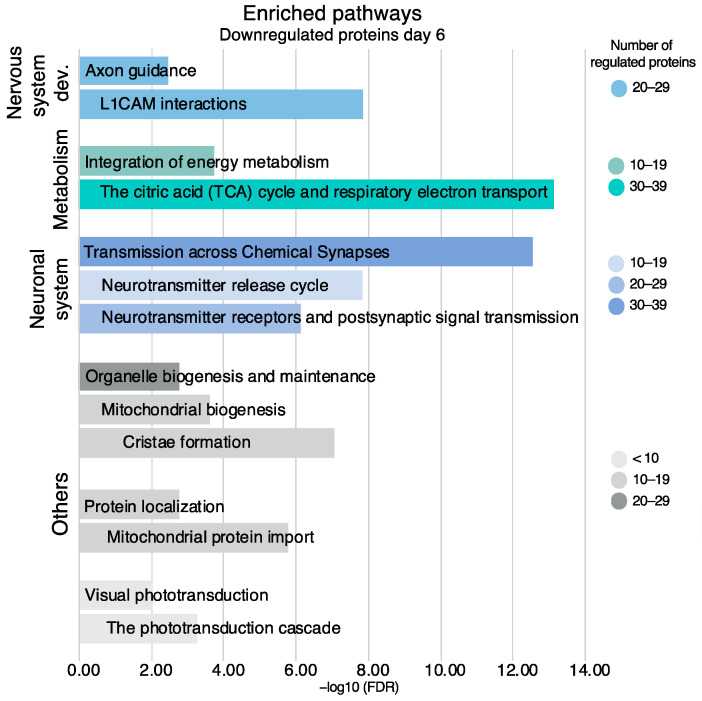
Enriched pathways and their sub-pathways based on all downregulated proteins on day six, non-exhaustive. Pathways are presented in accordance with the Reactome hierarchical organization. Coloring refers to the number of regulated proteins, the *x*-axis is the statistical significance expressed as −log_10_(FDR).

For the downregulated proteins on day six, the enrichment of several pathways in the neuronal system was evident (Figure 9). Pathways in the downregulated proteins included transmission across chemical synapses (37 proteins, FDR 2.95 × 10^−13^), neurotransmitter receptors and postsynaptic signal transmission (23 proteins, FDR 7.55 × 10^−7^), and neurotransmitter release cycle (14 proteins, FDR 1.43 × 10^−8^). The neurotransmitter release cycle included the proteins synaptotagmin-1 (fold change = 0.72) and complexin-1 (fold change = 0.50), while neurofilament light polypeptide (fold change = 0.79) was included in transmission across chemical synapses.

Other enriched pathways in the downregulated proteins included the citric acid (TCA) cycle and respiratory electron transport (39 proteins, FDR 7.37 × 10^−14^) and the phototransduction cascade (7 proteins, FDR 5.51 × 10^−4^). All the proteins in these pathways are listed in Table S10.

### 2.5. Immunohistochemistry

**Figure 10:**
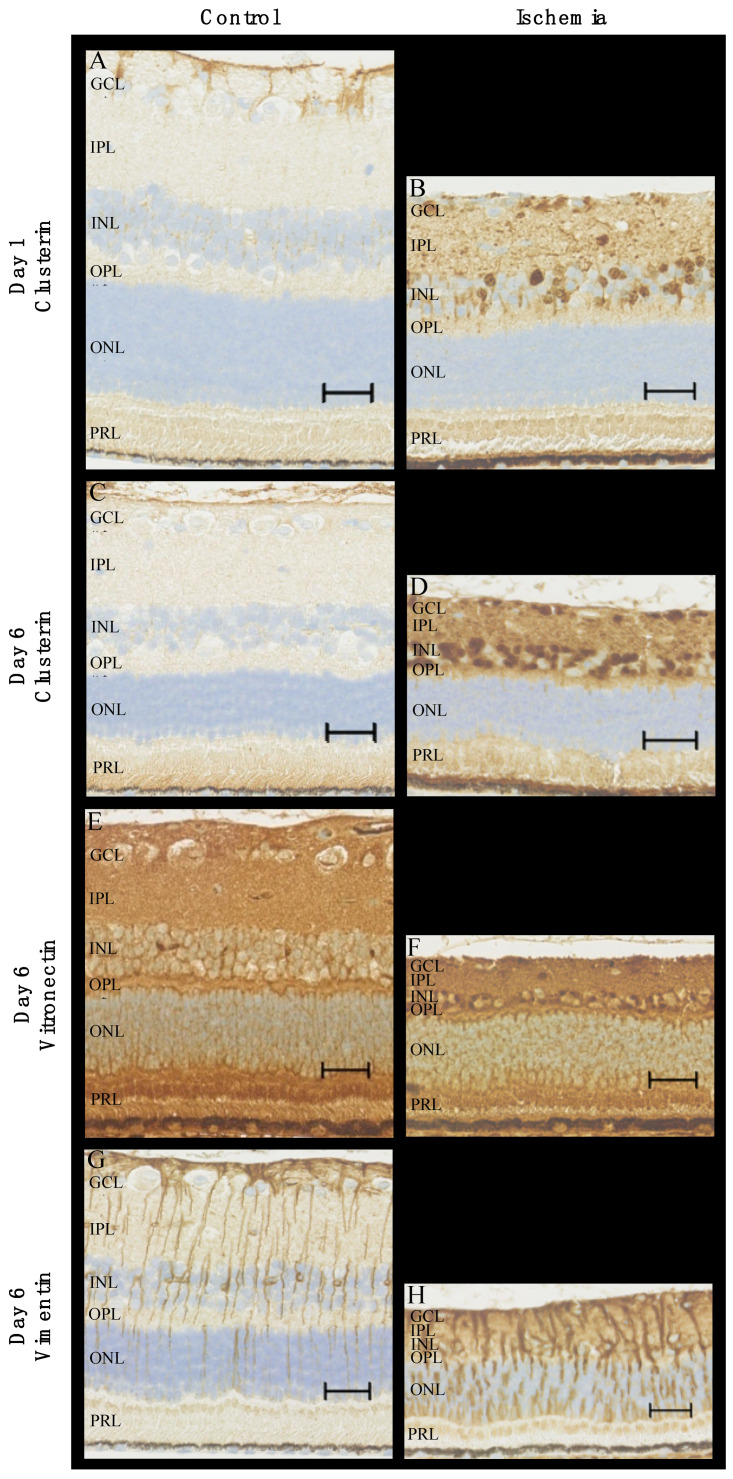
Immunohistochemical staining of ischemic retinas due to RAO and control retinas. (**A**–**D**) In RAO, an increased expression of clusterin was observed in the inner retinal layers on days one and six. More specifically, RAO was associated with increased levels of clusterin in the nuclei of the ganglion cell layer and the inner nuclear layer. (**E**,**F**) The content of vitronectin on day six was elevated in the inner retinal layers with strong expression in the nuclei of the inner nuclear layer. (**G**,**H**) Staining for vimentin (day six) with strong expression in Müller cells in the ischemic retinas. Pronounced gliosis in retinal Müller cells was observed in RAO. Scale bar 30 μm. GCL = ganglion cell layer, IPL = inner plexiform layer, INL = inner nuclear layer, OPL = outer plexiform layer, ONL = outer nuclear layer, PRL = photoreceptor layer.

For validation, clusterin, vitronectin, and vimentin were investigated with immunohistochemistry (Figure 10). Clusterin was tested on both days one and six, while vitronectin and vimentin were tested on day six only as these were not identified as upregulated on day one by the LC-MS/MS. In RAO, the staining for clusterin was stronger in the inner retinal layers (ganglion cell layer, inner plexiform layer, inner nuclear layer, outer plexiform layer) compared to the control, and staining was stronger on day six compared to day one. On day six, an increased expression of vitronectin was observed in the inner nuclear layer and, to a lesser extent, in the inner plexiform and ganglion cell layer of the ischemic retinas with RAO. RAO was associated with a strong expression of vimentin in the retinal Müller cells on day six.

Furthermore, the retinas with artery occlusion showed clear signs of ischemia with pronounced retinal atrophy and the disruption of the architecture of inner retinal layers, which were most prominent on day six compared to day one.

## 3. Discussion

We presented a model of successfully induced RAO in pigs using a standard argon laser. LC-MS/MS revealed differentially expressed proteins one, three, and six days after occlusion, and the number of proteins increased considerably during this time period. Enriched pathways on day six included immune system pathways and cellular response to stress.

Occlusions were validated by fundus photography and FA, both of which showed typical signs of RAO with ischemia. Our findings are very similar to clinical observations in patients with RAO and in previous animal models [12,13,14]. Some reperfusion and retrograde filling began to appear on day five—long after irreversible damage had occurred and in line with the clinical natural history of RAO [15,16]. In other studies using almost the same model, Goldenberg-Cohen et al. [10] and Kramer et al. [7] induced RAO by a laser combined with the photosensitizing agent Rose Bengal and found reperfusion to occur after only six hours. The various existing animal models of RAO have been discussed thoroughly elsewhere [11]. The advantages of the model chosen in this study include the similarity of pigs with humans, the occlusion of the retinal artery only without the pressure-induced damage of any structures, a noninvasive procedure, and the absence of dye that is less suited for proteomics.

In this study, the differences in proteins were most pronounced on day six, more subtle on day three, and especially on day one. As was evident from the immunohistochemical staining for clusterin and the LFQ values of several proteins, the protein levels most likely increased over time after the occlusion. Alternatively, the pronounced protein changes observed on day six, as opposed to days one and three, may potentially be driven by reperfusion damages. Interestingly, a previous study found only very limited changes in their short ischemic period, but after one and seven days of subsequent reperfusion, a large number of differentially expressed genes were identified [17]. The authors hypothesized that many of the observed changes in an ischemia/reperfusion model were oxygen/energy-dependent, and indeed, it is often the reperfusion rather than ischemia itself that causes much of the damage [17,18]. The majority of previous experimental studies are based on a short duration of ischemia, often followed by reperfusion. In that set-up, the studied molecular changes may be caused by reperfusion, but it might very well be the changes taking place during the ischemic phase that is most relevant in retinal artery occlusion as irreversible damage and subsequent blindness of the retina has already occurred at the time of reperfusion [11].

The pathway analysis of a large number of differentially expressed proteins on day six confirmed the upregulation of proteins involved in hemostasis which would be expected in a successful model of artery occlusion. Other enriched pathways included the innate immune system and cytokine signaling in immune system processes herein, such as IL-1 and IL-12 signaling. The activation of the immune system is seen in ischemia/reperfusion damage in general and has received much attention within the field of cerebral ischemia, including the role of cytokines and the neuroprotective potential of immunomodulation [19]. For instance, the potential of IL-1 receptor antagonists has been investigated [19].

Immune system activation has also been identified in retinal ischemia. Although different from those shown in our study, Kramer et al. [7] demonstrated an increase in the proinflammatory cytokines TNF-α, IL-6, and MIP-2 (murine equivalent to IL-8) within the first day and normalizing at day seven in their animal study on laser-induced central RAO in mice. The source of this peak of protein changes after a few hours, as opposed to six days in the present study, may be the relatively short ischemic period of only six hours.

The enrichment analysis also showed a downregulation in pathways in the neuronal system, such as transmission across chemical synapses. A previous proteomic study by Tian et al. [20] used a mouse model based on high intraocular pressure with only 60 min of ischemia and also found synapse-related protein networks to be downregulated, including the protein synaptotagmin-1 when simultaneous retinal atrophy was evident. Likewise, synaptotagmin-1 was also downregulated in our study, as were complexin-1 and neurofilament light polypeptide, which was also involved in transmission across chemical synapses and found in previous studies from our group on retinal vein occlusion [21,22]. The downregulation of synaptotagmin-1, complexin-1, and neurofilament light polypeptide is likely to reflect degenerative changes following ischemia and is in line with the observed downregulation of the phototransduction cascade. It is worth noting that the study by Tian et al. [20] additionally found annexin A1, GFAP, and vimentin to be upregulated, which is consistent with our findings.

We found both vimentin and GFAP to be upregulated (by fold-change 2.26 and 1.98, respectively), and vimentin was validated by IHC. Vimentin and GFAP are both intermediate filaments and are well known to be upregulated in Müller cells and astrocytes in general in the CNS as a response to stress, for instance, ischemia [23,24,25,26]. More specifically, elevated levels of vimentin and GFAP have previously been identified in a pig model of ischemic reperfusion induced by high intraocular pressure [23].

Clusterin was upregulated on days one and six and validated by IHC. Clusterin acts as a chaperon, relates to proteostasis (protein homeostasis), and is linked to protective mechanisms associated with the survival of the cell and organism [27]. It has been shown to be associated with a wide range of diseases, including ophthalmic diseases such as retinal vein occlusion, diabetic retinopathy, and age-related macular degeneration [6]. In addition, clusterin has previously been shown to be upregulated in ischemic tissue, including the retina, although the latter seems to have received less attention [28].

Likewise, vitronectin has also been found to be increased in cerebral ischemia [29]. Vitronectin is predominantly produced in the liver and can be released into the bloodstream. In the case of acute tissue injury, it can contribute to thrombus formation, the stability of vessel occlusion, and the following immune and inflammatory responses [30]. A recent study on induced strokes in mice found the leakage of vitronectin into the brain to be detrimental but in female mice only [31].

Numerous of the most upregulated proteins and those included in the major cluster in our study were found in previous studies from our group on retinal vein occlusion, which also resulted in retinal ischemia. Proteins that were upregulated in our study and in experimental retinal vein occlusion included fibronectin, annexin A1, galectin-3, alpha-crystallin B chain, vimentin, annexin A2, STAT1, GFAP, osteopontin, vitronectin, and clusterin [21,22]. Specifically, aqueous fibronectin was recently found to correlate with the severity of macular edema and visual acuity in patients with branch retinal vein occlusion [32]. Further studies are encouraged to elucidate the significance of these proteins in RAO and retinal ischemia in general and further on the potential of future treatment targets.

The regulated proteins identified by mass spectrometry were not corrected for multiple hypothesis testing; however, the *p*-value was lowered to 0.01. As the study was based on the discovery of proteomics in a new model, the priority was to minimize the risk of type two errors. Furthermore, we judged that a minority of false positives would not significantly change the findings of important biochemical pathways [33].

The pathway analysis was carried out separately for the up and down-regulated proteins due to their involvement in different pathways, and a combined analysis was, therefore, less clear; however, it did not result in drastically different results.

The more subtle changes in proteins on day one could potentially be further investigated with more power, i.e., the use of more animals. However, the use of animals is always a balancing of potential benefits and ethical considerations, especially as pigs are large and expensive animals.

## 4. Materials and Methods

### 4.1. Animals

Thirteen female Danish Landrace pigs of approximately 20 kg were used for this experiment. All animals were anesthetized with an intramuscular injection of 5 mL Zoletil 50 Vet (a mixture of tiletamine 6.25 mg/mL and zolazepam 6.25 mg/mL; Virbac, Carros, France), ketamine 6.25 mg/mL (Ketaminol Vet; MSD, Rahway, NJ, USA), butorphanol 1.25 mg/mL (Dolorex; MSD, Rahway, NJ, USA), and xylain 6.25 mg/mL (Rompun Vet; Bayer, Leverkusen, Germany. The eyes were anesthetized with oxybuprocaine hydrochloride 0.4% (Bausch & Lomb, Rochester, NY, USA) and tetracaine 1% (Bausch & Lomb, Rochester, NY, USA), followed by dilatation with tropicamide 0.5% (Mydriacyl; Bausch & Lomb, Rochester, NY, USA) and phenylephrine 10% (Metaoxidrin; Bausch & Lomb, Rochester, NY, USA). To prevent the corneal surface from drying and, thereby, compromising the view of the retina, Systane Ultra eye drops (Polyethylene Glycol 400, Propylene Glycol; Alcon, Copenhagen, Denmark) were applied regularly. This study was approved by the Danish Animal Experiments Inspectorate (permission number 2019-15-0201-01651).

### 4.2. Induction of RAO

In each animal, RAO was induced in the right eye using a standard argon laser (532 nm), which was applied by indirect ophthalmoscopy directly on the superior branch retinal artery adjacent to the optic disc. The laser was applied until the stagnation of retinal blood flow and paleness consistent with retinal ischemia appeared. Approximately 200 applications with an exposure of 200–300 ms were used for each occlusion. The power gradually increased to 400–600 mW. In the left eye, which served as a control, an identical area of laser applications was created in a corresponding area but without major vessels.

### 4.3. Validation of RAO

Experimental RAO was considered successful when indirect ophthalmoscopy showed the formation of thrombotic material, the segmentation of the blood column in the artery, and paleness of the retina supplied by the occluded branch artery, which was consistent with retinal ischemia. In all pigs, this occlusion was further validated by fluorescein angiography (FA) (Heidelberg fluorescein angiography and RETI-map-animal, Roland Consult, Berlin, Germany) and fundus photographs (Optomed Aurora, Oulo, Finland) prior to enucleation or the day before.

### 4.4. Sample Preparation

For proteomic analysis, eyes with RAO were collected on day one (*n* = 4), day three (*n* = 2), and day six (*n* = 5), along with the control eyes (*n* = 11). The animals were euthanized immediately after enucleation. The eyes were placed on ice, and the globes were opened by incisions into the sclera two mm posterior to the limbus. The anterior compartment and the vitreous body were removed. For LC-MS/MS, retinas were peeled from the eyecup, placed in an Eppendorf tube, and stored at −80 °C until further use.

### 4.5. Mass Spectrometry

#### 4.5.1. Preparation of Samples for Proteomic Analysis

Twenty-two pig retinal samples (11 controls and eyes with RAO, including four from day one, two from day three, and five from day six) were prepared for proteomic analysis using the suspension trapping (STrap) method [34]. In short, the retinal tissue was dissolved in a 600 µL lysis buffer (5% SDS, 50 mM TEAB) and sonicated on ice. After incubation for 5 min. at 99 °C with agitation (600 rpm), samples were centrifuged at 16,000× *g* for 10 min, and the protein concentration was measured by infrared spectrometry as previously described [35]. One hundred µg of each sample was processed with S-Trap mini spin columns from Protifi (Huntington, NY, USA) following the protocol as described by the manufacturer. Alkylation was performed using TCEP and iodoacetamide before tryptic digestion, which was performed overnight at 37 °C in a wet chamber. Peptides were then eluded from the columns and quantified by fluorescence as described [35]. Finally, the peptides were dissolved at a concentration of 1 µg/µL in 0.1% formic acid.

#### 4.5.2. Liquid Chromatography—Tandem Mass Spectrometry

Analyses were performed by injecting 2 µg in quadruplicate into the mass spectrometry platform consisting of an Ultimate 3000 nano LC connected to an Orbitrap Fusion Tribrid MS (Thermo Fisher Scientific, Waltham, MA, USA). Peptides were eluted over two hours by mixing buffer A (99.9% water, 0.1% formic acid) with increasing concentrations of buffer B (99.9% acetonitrile, 0.1% formic acid). This universal method was used with the settings described [36].

A total of 88 raw data files were entered into MaxQuant version 1.6.6.0 for LFQ analysis [37] and searched against the Uniprot *Sus scrofa* database and the *Homo sapiens* database downloaded on 8 November 2020. Generally, the default settings were used in MaxQuant, including a false discovery rate (FDR) of 0.01 for protein identification and peptide spectrum matches. Digestion with Trypsin was used instead of Trypsin/P, an LFQ minimum ratio count was set to 1, and the match between the runs function was used.

#### 4.5.3. Statistical Analysis

Data were further processed with Perseus version 1.6.14.0 (Max Planck Institute of Biochemistry, Martinsried, Germany) [38] and filtered from potential contaminants, proteins identified in the reverse database, and proteins only identified by post-translational modifications. At least two unique peptides were required for identification. Data were log_2_ transformed, and the mean of the valid values in the technical replica was used as the protein level in each biological sample. Only proteins identified in at least 70% of the biological samples within each group were included. The median coefficient of variation in the proteins in each sample ranged between 13.7% and 19.9%, with a mean of 16.2%. Student’s *t*-test was used for the statistical analysis of protein changes. Proteins were considered significantly regulated if *p* < 0.01.

Unsupervised PCA was obtained through Perseus to assess trends in proteome changes throughout the different days and was compared to the control samples. Prior to PCA, data were filtered to include proteins that could be identified in all samples. Volcano plots were produced in Perseus. Scatter plots of the LFQ intensity levels for selected proteins were made in GraphPad Prism 9.5.1 (GraphPad Software, La Jolla, CA, USA).

#### 4.5.4. Bioinformatic Analyses

Regulated proteins on day six were selected for further bioinformatic analyses. The protein–protein interaction network and cluster analysis were performed in STRING version 11.5 [39] for proteins with a fold-change > 1.75. The UniProt *Homo sapiens* accession number was used, and the minimum required interaction score was set to 0.400. The network was clustered by MCL using an inflation parameter of 2.5. Functional enrichment analyses were performed in Reactome (Pathway Browser version 3.7, database release 83) [40]. Separate analyses were carried out for all the up and downregulated proteins. Based on these data, a bar chart was made in Microsoft Excel version 16.7 and Adobe Illustrator version 23.0.2.

### 4.6. Immunohistochemistry

Immunohistochemistry was performed to compare RAO at day one (*n* = 1) vs. control (*n* = 1) and RAO at day six (*n* = 1) vs. the control (*n* = 1). Complexes comprised the retina, choroid, and sclera were immersed in a fixative solution containing 750 mL 0.1 M phosphate buffer, 100 mL formalin, and 150 mL distillated water for 12 h at 4 °C. Hereafter, the retinas were stored in a phosphate buffer pH 7.4 at 4 °C until further use. Immunohistochemistry was performed as previously described [21] with a mouse monoclonal IgG anti-vimentin antibody (AB8069, Abcam, Cambridge, UK) 1:2000, a rabbit polyclonal anti-clusterin antibody (MyBiosource, San Diego, CA, USA) 1:400, and a mouse monoclonal IgG anti-vitronectin antibody (66398-1-Ig, Proteintech, Manchester, UK) 1:400.

## 5. Conclusions

The presented animal model proved suitable for the proteomic analyses of RAO. RAO was successfully induced, lasted for several days, and resulted in the regulation of numerous proteins as identified by LC-MS/MS, especially on day six. Of these, various proteins have been previously linked to ischemia, including clusterin, vimentin, and vitronectin. Furthermore, several enriched pathways corresponded well with a reaction to RAO, including cellular response to stress, hemostasis, innate immune response, and cytokine signaling, as well as the downregulation of proteins involved in transmission across chemical synapses and visual phototransduction.

## Figures and Tables

**Table 1:** Upregulated proteins with fold-change > 1.75 on day six detected by LC-MS/MS, ordered according to fold-change.

Fold Change	Protein ID	Protein Name	Gene Name	*p*-Value	Upregulated on Other Timepoints
83.56	Q14315-2	Filamin-C	FLNC	<0.00001	Day 1 + Day 3
14.70	P13796	Plastin-2	LCP1	0.00000	
14.42	P14287	Osteopontin	SPP1	<0.00001	Day 3
11.91	P48819	Vitronectin	VTN	0.00043	
10.10	Q14764	Major vault protein	MVP	<0.00001	
8.79	P02751-1	Fibronectin	FN1	0.00024	
8.18	P06867	Plasminogen	PLG	0.00131	
7.41	P00915	Carbonic anhydrase 1	CA1	0.00487	
6.14	P02675	Fibrinogen beta chain	FGB	0.00123	
5.80	P27594	Interferon-induced GTP-binding protein Mx1	MX1	0.00147	
5.17	A7VK00	Interferon-induced GTP-binding protein Mx2	MX2	0.00050	
5.12	P02679-2	Fibrinogen gamma chain	FGG	0.00045	
4.29	P19133	Ferritin light chain	FTL	0.00001	
4.23	Q29549	Clusterin	CLU	<0.00001	Day 1 + Day 3
3.67	O75369-2	Filamin-B	FLNB	<0.00001	
3.65	P00450	Ceruloplasmin	CP	0.00406	
3.46	P18650	Apolipoprotein E	APOE	<0.00001	
3.39	P02067	Hemoglobin subunit beta	HBB	0.00010	Day 1 + Day 3
3.07	O62839	Catalase	CAT	0.00171	
3.01	Q6P1M3	Lethal(2) giant larvae protein homolog 2	LLGL2	<0.00001	Day 3
2.91	Q764M5	Signal transducer and activator of transcription 1	STAT1	0.00001	
2.82	O15143	Actin-related protein 2/3 complex subunit 1B	ARPC1B	0.00014	Day 3
2.81	Q19S50	Signal transducer and activator of transcription 3	STAT3	<0.00001	Day 3
2.69	P01965	Hemoglobin subunit alpha	HBA1	0.00083	
2.68	Q15417	Calponin-3	CNN3	0.00003	
2.65	P19620	Annexin A2	ANXA2	<0.00001	
2.62	Q7M2W6	Alpha-crystallin B chain	CRYAB	0.00002	Day 3
2.61	Q29095	Prostaglandin-H2 D-isomerase	PTGDS	0.00048	
2.58	P17931	Galectin-3	LGALS3	0.00001	
2.55	Q8MJ14	Glutathione peroxidase 1	GPX1	0.00834	
2.50	P19619	Annexin A1	ANXA1	<0.00001	Day 1 + Day 3
2.50	Q96TA1-2	Niban-like protein 1	FAM129B	0.00004	
2.39	P18648	Apolipoprotein A1	APOA1	0.00259	
2.37	P50447	Alpha-1-antitrypsin	SERPINA1	0.00874	
2.35	P02794	Ferritin heavy chain	FTH1	0.00106	
2.28	P37802	Transgelin-2	TAGLN2	<0.00001	Day 3
2.27	P29700	Alpha-2-HS-glycoprotein	AHSG	0.00990	
2.26	P02543	Vimentin	VIM	<0.00001	Day 3
2.25	P08758	Annexin A5	ANXA5	<0.00001	
2.23	Q96CX2	BTB/POZ domain-containing protein KCTD12	KCTD12	0.00020	
2.20	Q9BUF5	Tubulin beta-6 chain	TUBB6	0.00521	
2.17	P21333-2	Filamin-A	FLNA	<0.00001	Day 3
2.12	Q9BX66-9	Sorbin and SH3 domain-containing protein 1	SORBS1	0.00002	
2.11	Q96HF1	Secreted frizzled-related protein 2	SFRP2	<0.00001	Day 3
2.09	Q9H223	EH domain-containing protein 4	EHD4	0.00017	
2.02	O00161	Synaptosomal-associated protein 23	SNAP23	0.00038	
1.99	P46940	Ras GTPase-activating-like protein IQGAP1	IQGAP1	<0.00001	
1.98	P14136-3	Glial fibrillary acidic protein	GFAP	0.00010	
1.98	Q4FAT7	Beta-glucuronidase	GUSB	0.00525	
1.97	O00299	Chloride intracellular channel protein 1	CLIC1	<0.00001	Day 3
1.94	P40121	Macrophage-capping protein	CAPG	<0.00001	
1.92	P27105	Erythrocyte band 7 integral membrane protein	STOM	0.00128	
1.92	P04439-2	HLA class I histocompatibility antigen, A-3 alpha chain	HLA-A	0.00907	
1.91	Q5GLZ8-6	Probable E3 ubiquitin-protein ligase HERC4	HERC4	0.00045	
1.91	P21980-3	Protein-glutamine gamma-glutamyltransferase 2	TGM2	0.00415	
1.89	O77564	Glutamate carboxypeptidase 2	FOLH1	0.00466	
1.87	P84095	Rho-related GTP-binding protein RhoG	RHOG	0.00114	
1.86	Q06210-2	Glutamine--fructose-6-phosphate aminotransferase [isomerizing] 1	GFPT1	0.00918	
1.85	O15511	Actin-related protein 2/3 complex subunit 5	ARPC5	0.00003	Day 3
1.85	Q96KP4-2	Cytosolic non-specific dipeptidase	CNDP2	<0.00001	
1.84	Q14254	Flotillin-2	FLOT2	0.00001	Day 3
1.84	Q29099	Polypyrimidine tract-binding protein 1	PTBP1	0.00006	
1.84	Q9UNF1-2	Melanoma-associated antigen D2	MAGED2	0.00026	
1.84	Q5TZA2	Rootletin	CROCC	<0.00001	
1.82	P30043	Flavin reductase (NADPH)	BLVRB	0.00043	Day 3
1.82	Q863Z0	Proteasome activator complex subunit 2	PSME2	0.00571	
1.82	P62495-2	Eukaryotic peptide chain release factor subunit 1	ETF1	0.00009	
1.80	Q09666	Neuroblast differentiation-associated protein AHNAK	AHNAK	0.00003	
1.80	P09571	Serotransferrin	TF	0.00407	
1.78	Q5PXT2	LIM and cysteine-rich domains protein 1	LMCD1	0.00044	
1.77	Q64L94	Proteasome activator complex subunit 1	PSME1	<0.00001	
1.77	Q29116-2	Tenascin	TNC	0.00977	
1.77	Q6NZI2	Polymerase I and transcript release factor	PTRF	0.00001	
1.76	P09493-10	Tropomyosin alpha-1 chain	TPM1	0.00008	

## Data Availability

Please refer to the Supplementary Materials.

## Supplementary Materials

The following supporting information can be downloaded at: https://www.mdpi.com/article/10.3390/ijms24097919/s1.

## References

[1] Tobalem S., Schutz J.S., Chronopoulos A. (2018). Central retinal artery occlusion—Rethinking retinal survival time. BMC Ophthalmol..

[2] Rudkin A.K., Lee A.W., Aldrich E., Miller N.R., Chen C.S. (2010). Clinical characteristics and outcome of current standard management of central retinal artery occlusion. Clin. Experiment. Ophthalmol..

[3] Dattilo M., Biousse V., Newman N.J. (2017). Update on the management of central retinal artery occlusion. Neurol. Clin..

[4] Osborne N.N., Casson R.J., Wood J.P.M., Chidlow G., Graham M., Melena J. (2004). Retinal ischemia: Mechanisms of damage and potential therapeutic strategies. Prog. Retin. Eye Res..

[5] Nguyen D.D., Luo L.J., Yang C.J., Lai J.Y. (2023). Highly Retina-Permeating and Long-Acting Resveratrol/Metformin Nanotherapeutics for Enhanced Treatment of Macular Degeneration. ACS Nano.

[6] Cehofski L.J., Honoré B., Vorum H. (2017). A review: Proteomics in retinal artery occlusion, retinal vein occlusion, diabetic retinopathy and acquired macular disorders. Int. J. Mol. Sci..

[7] Kramer M., Dadon S., Hasanreisoglu M., Monselise Y., Avraham B.R., Feldman A., Eldar I., Weinberger D., Goldenberg-Cohen N. (2009). Proinflammatory cytokines in a mouse model of central retinal artery occlusion. Mol. Vis..

[8] Kramer M., Goldenberg-Cohen N., Axer-Siegel R., Weinberger D., Cohen Y., Monselise Y. (2005). Inflammatory reaction in acute retinal artery occlusion: Cytokine levels in aqueous humor and serum. Ocul. Immunol. Inflamm..

[9] Zhang Y., Cho C.H., Atchaneeyasakul L.O., McFarland T., Appukuttan B., Stout J.T. (2005). Activation of the mitochondrial apoptotic pathway in a rat model of central retinal artery occlusion. Investig. Ophthalmol. Vis. Sci..

[10] Goldenberg-Cohen N., Dadon S., Avraham B.C.R., Kramer M., Hasanreisoglu M., Eldar I., Weinberger D., Bahar I. (2008). Molecular and histological changes following central retinal artery occlusion in a mouse model. Exp. Eye Res..

[11] Vestergaard N., Cehofski L.J., Honoré B., Aasbjerg K., Vorum H. (2019). Animal models used to simulate retinal artery occlusion: A comprehensive review. Transl. Vis. Sci. Technol..

[12] Beatty S., Au Eong K.G. (2000). Acute occlusion of the retinal arteries: Current concepts and recent advances in diagnosis and management. J. Accid. Emerg. Med..

[13] Isono H., Kishi S., Kimura Y., Hagiwara N., Konishi N., Fujii H. (2003). Observation of choroidal circulation using index of erythrocytic velocity. Arch. Ophthalmol. (Chic. Ill 1960).

[14] Vine A.K., Maguire P.T., Martonyi C., Kincaid M.C. (1988). Recombinant tissue plasminogen activator to lyse experimentally induced retinal arterial thrombi. Am. J. Ophthalmol..

[15] Hayreh S.S., Zimmerman M.B., Kimura A., Sanon A. (2004). Central retinal artery occlusion. Retinal survival time. Exp. Eye Res..

[16] Hayreh S.S. (2011). Acute retinal arterial occlusive disorders. Prog. Retin. Eye Res..

[17] Andreeva K., Zhang M., Fan W., Li X., Chen Y., Rebolledo-Mendez J.D., Cooper N.G. (2014). Time-dependent gene profiling indicates the presence of different phases for ischemia/reperfusion injury in retina. Ophthalmol. Eye Dis..

[18] White B.C., Sullivan J.M., DeGracia D.J., O’Neil B.J., Neumar R.W., Grossman L.I., Rafols J.A., Krause G.S. (2000). Brain ischemia and reperfusion: Molecular mechanisms of neuronal injury. J. Neurol. Sci..

[19] Jiang Q., Stone C.R., Elkin K., Geng X., Ding Y. (2021). Immunosuppression and neuroinflammation in stroke pathobiology. Exp. Neurobiol..

[20] Tian H., Wang L., Cai R., Zheng L., Guo L. (2014). Identification of protein network alterations upon retinal ischemia-reperfusion injury by quantitative proteomics using a Rattus norvegicus model. PLoS ONE.

[21] Cehofski L.J., Kruse A., Alsing A.N., Sejergaard B.F., Nielsen J.E., Schlosser A., Sorensen G.L., Grauslund J., Honoré B., Vorum H. (2022). Proteome analysis of aflibercept intervention in experimental central retinal vein occlusion. Molecules.

[22] Cehofski L.J., Kruse A., Kirkeby S., Alsing A.N., Ellegaard Nielsen J., Kojima K., Honoré B., Vorum H. (2018). IL-18 and S100A12 are upregulated in experimental central retinal vein occlusion. Int. J. Mol. Sci..

[23] Wurm A., Iandiev I., Uhlmann S., Wiedemann P., Reichenbach A., Bringmann A., Pannicke T. (2011). Effects of ischemia-reperfusion on physiological properties of Müller glial cells in the porcine retina. Investig. Ophthalmol. Vis. Sci..

[24] Bringmann A., Pannicke T., Grosche J., Francke M., Wiedemann P., Skatchkov S.N., Osborne N.N., Reichenbach A. (2006). Müller cells in the healthy and diseased retina. Prog. Retin. Eye Res..

[25] Hol E.M., Pekny M. (2015). Glial fibrillary acidic protein (GFAP) and the astrocyte intermediate filament system in diseases of the central nervous system. Curr. Opin. Cell Biol..

[26] Gesslein B., Håkansson G., Carpio R., Gustafsson L., Perez M.T., Malmsjö M. (2010). Mitogen-activated protein kinases in the porcine retinal arteries and neuroretina following retinal ischemia-reperfusion. Mol. Vis..

[27] Wilson M.R., Zoubeidi A. (2017). Clusterin as a therapeutic target. Expert Opin. Ther. Targets.

[28] Gwon J.S., Kim I.B., Lee M.Y., Oh S.J., Chun M.H. (2004). Expression of clusterin in Müller cells of the rat retina after pressure-induced ischemia. Glia.

[29] del Zoppo G.J., Milner R., Mabuchi T., Hung S., Wang X., Berg G.I., Koziol J.A. (2007). Microglial activation and matrix protease generation during focal cerebral ischemia. Stroke.

[30] Leavesley D.I., Kashyap A.S., Croll T., Sivaramakrishnan M., Shokoohmand A., Hollier B.G., Upton Z. (2013). Vitronectin—Master controller or micromanager?. IUBMB Life.

[31] Jia C., Malone H.M., Keasey M.P., Lovins C., Elam J., Hagg T. (2020). Blood vitronectin induces detrimental brain interleukin-6 and correlates with outcomes after stroke only in female mice. Stroke.

[32] Cehofski L.J., Kojima K., Terao N., Kitazawa K., Thineshkumar S., Grauslund J., Vorum H., Honoré B. (2020). Aqueous fibronectin correlates with severity of macular edema and visual acuity in patients with branch retinal vein occlusion: A proteome study. Investig. Ophthalmol. Vis. Sci..

[33] Diz A.P., Carvajal-Rodríguez A., Skibinski D.O.F. (2011). Multiple hypothesis testing in proteomics: A strategy for experimental work. Mol. Cell. Proteom..

[34] Zougman A., Selby P.J., Banks R.E. (2014). Suspension trapping (STrap) sample preparation method for bottom-up proteomics analysis. Proteomics.

[35] Honoré B. (2020). Proteomic protocols for differential protein expression analyses. Methods Mol. Biol..

[36] Ludvigsen M., Thorlacius-Ussing L., Vorum H., Moyer M.P., Stender M.T., Thorlacius-Ussing O., Honoré B. (2020). Proteomic characterization of colorectal cancer cells versus normal-derived colon mucosa cells: Approaching identification of novel diagnostic protein biomarkers in colorectal cancer. Int. J. Mol. Sci..

[37] Tyanova S., Temu T., Cox J. (2016). The MaxQuant computational platform for mass spectrometry-based shotgun proteomics. Nat. Protoc..

[38] Tyanova S., Temu T., Sinitcyn P., Carlson A., Hein M.Y., Geiger T., Mann M., Cox J. (2016). The Perseus computational platform for comprehensive analysis of (prote)omics data. Nat. Methods.

[39] Szklarczyk D., Gable A.L., Nastou K.C., Lyon D., Kirsch R., Pyysalo S., Doncheva N.T., Legeay M., Fang T., Bork P. (2021). The STRING database in 2021: Customizable protein–protein networks, and functional characterization of user-uploaded gene/measurement sets. Nucleic Acids Res..

[40] Gillespie M., Jassal B., Stephan R., Milacic M., Rothfels K., Senff-Ribeiro A., Griss J., Sevilla C., Matthews L., Gong C. (2022). The reactome pathway knowledgebase 2022. Nucleic Acids Res..

