# Supplementary material for: Large-Scale Protein Analysis of Experimental Retinal Artery Occlusion
Source: Int J Mol Sci. 2023 Apr 27;24(9):7919. doi: 10.3390/ijms24097919 (PMC10177937; doi:10.3390/ijms24097919)
Supplement: Supplementary file 1 [file ijms-24-07919-s001.zip › Figure S1, FA from all animals.pdf]

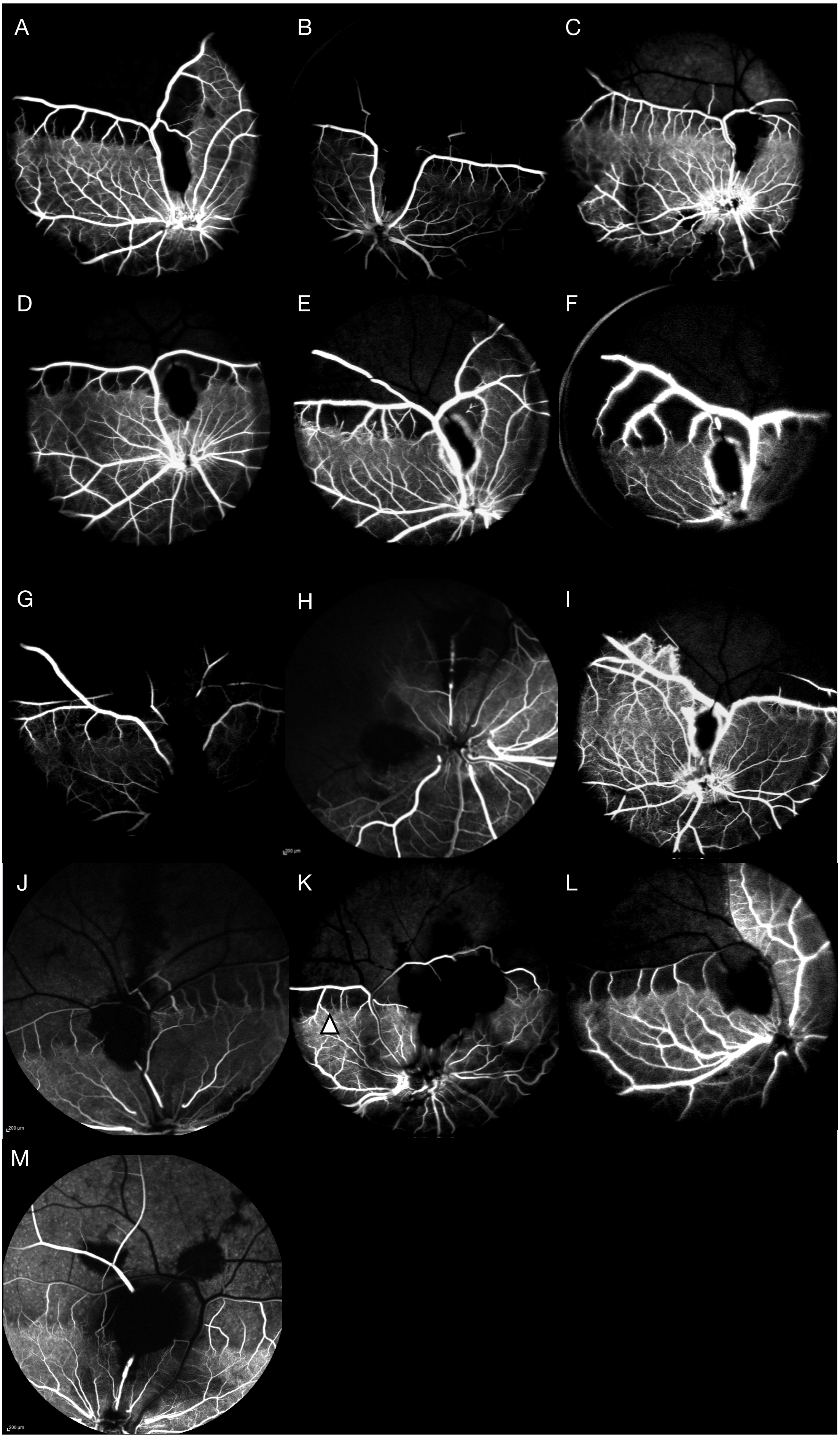

Supplementary figure1: Presentation of a flourescein angiography image from each animal in the study. A: Day 1, 14s; B: Day 3, 22s; C: Day 3, 20s; D: Day 1, 23s; E: Day 1, 23s; F: Day 1, 70s; G: Day 3, 30s; H: Day 5, 9s; I: day 1, 25s; J: day 5, 8s; K: Day 2, 15s; L: Day 1, 11s; M: Day 5, 22s.
