# Supplementary material for: Large-Scale Protein Analysis of Experimental Retinal Artery Occlusion
Source: Int J Mol Sci. 2023 Apr 27;24(9):7919. doi: 10.3390/ijms24097919 (PMC10177937; doi:10.3390/ijms24097919)
Supplement: Supplementary file 1 [file ijms-24-07919-s001.zip › Figure S2, control eye.pdf]

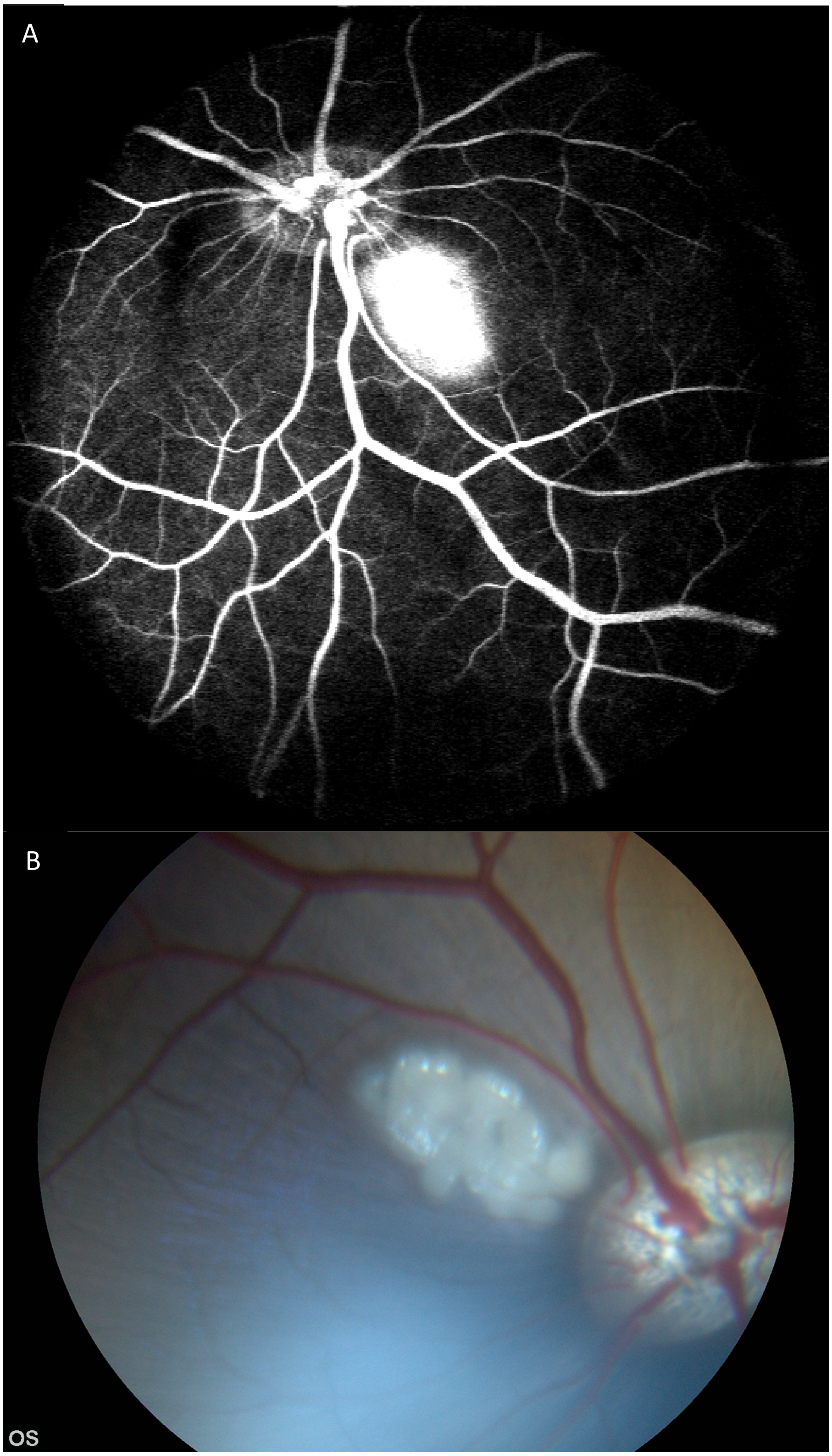

Supplementary figure 2: A) Flourescein angiography from control eye showing sham laser spot and normal filling of the vessels. B) Fundus photo from control eye showing sham laser spot but no retinal artery occlusion.
