# Supplementary material for: Large-Scale Protein Analysis of Experimental Retinal Artery Occlusion
Source: Int J Mol Sci. 2023 Apr 27;24(9):7919. doi: 10.3390/ijms24097919 (PMC10177937; doi:10.3390/ijms24097919)
Supplement: Supplementary file 1 [file ijms-24-07919-s001.zip › Table S10, Protein names in key pathways.pdf]

Selected upregulated pathways		
**Pathway name**	**Number of proteins**	**Submitted entities found**
Cellular response to stress	49	Cytoplasmic dynein 1 heavy chain 1<br>Flavin reductase<br>Thioredoxin<br>26S proteasome non-ATPase regulatory subunit 7<br>26S proteasome regulatory subunit 8<br>Apolipoprotein A-I<br>Catalase<br>40S ribosomal protein S10<br>MAP kinase-activated protein kinase 2<br>Hemoglobin subunit alpha<br>40S ribosomal protein S6<br>Protein disulfide-isomerase<br>Signal transducer and activator of transcription 3<br>60S ribosomal protein L18<br>26S proteasome non-ATPase regulatory subunit 11<br>60S ribosomal protein L4<br>Cytoplasmic dynein 1 intermediate chain 2<br>Glucose-6-phosphate 1-dehydrogenase<br>Glutamine--fructose-6-phosphate aminotransferase [isomerizing] 1<br>Tubulin beta-6 chain<br>Endoplasmic reticulum chaperone BiP<br>Elongation factor 1-alpha 1<br>40S ribosomal protein S8<br>Eukaryotic translation initiation factor 2 subunit 2<br>40S ribosomal protein S24<br>Proteasome subunit alpha type-6<br>Alpha-crystallin B chain<br>Glutathione peroxidase 1<br>40S ribosomal protein S12<br>Proteasome subunit alpha type-5<br>Proteasome activator complex subunit 1<br>60S ribosomal protein L35<br>26S proteasome regulatory subunit 10B<br>60S ribosomal protein L14<br>Protein disulfide-isomerase A6<br>Hemoglobin subunit beta<br>Proteasome subunit alpha type-4<br>Eukaryotic translation initiation factor 2 subunit 1<br>Glutamate--cysteine ligase catalytic subunit<br>Talin-1<br>Proteasome activator complex subunit 2<br>60S ribosomal protein L23<br>60S ribosomal protein L10a<br>Endoplasmin<br>Peroxiredoxin-1<br>Histone-binding protein RBBP4<br>F-actin-capping protein subunit alpha-1<br>Transcription factor p65<br>40S ribosomal protein S14
Cellular response to hypoxia<br>+<br>Oxygen-dependent proline hydroxylation of Hypoxia-inducible Factor Alpha	9	Proteasome activator complex subunit 2<br>26S proteasome regulatory subunit 10B<br>Proteasome subunit alpha type-5<br>26S proteasome regulatory subunit 8<br>Proteasome subunit alpha type-4<br>26S proteasome non-ATPase regulatory subunit 11<br>Proteasome activator complex subunit 1<br>Proteasome subunit alpha type-6<br>26S proteasome non-ATPase regulatory subunit 7
Cellular response to chemical stress	20	Proteasome activator complex subunit 2<br>Proteasome subunit alpha type-5<br>Peroxiredoxin-1<br>26S proteasome non-ATPase regulatory subunit 11<br>Thioredoxin<br>Flavin reductase<br>Proteasome activator complex subunit 1<br>Glucose-6-phosphate 1-dehydrogenase<br>26S proteasome non-ATPase regulatory subunit 7<br>26S proteasome regulatory subunit 10B<br>26S proteasome regulatory subunit 8<br>Hemoglobin subunit beta<br>Proteasome subunit alpha type-4<br>Catalase<br>Proteasome subunit alpha type-6<br>Hemoglobin subunit alpha<br>Glutamate--cysteine ligase catalytic subunit<br>Protein disulfide-isomerase<br>Glutathione peroxidase 1<br>Signal transducer and activator of transcription 3
KEAP1-NFE2L2 pathway	12	Proteasome activator complex subunit 2<br>26S proteasome regulatory subunit 10B<br>Proteasome subunit alpha type-5<br>26S proteasome regulatory subunit 8<br>Proteasome subunit alpha type-4<br>Peroxiredoxin-1<br>26S proteasome non-ATPase regulatory subunit 11<br>Proteasome activator complex subunit 1<br>Glucose-6-phosphate 1-dehydrogenase<br>Proteasome subunit alpha type-6<br>26S proteasome non-ATPase regulatory subunit 7<br>Glutamate--cysteine ligase catalytic subunit
Detoxification of Reactive Oxygen Species	5	Peroxiredoxin-1<br>Catalase<br>Thioredoxin<br>Protein disulfide-isomerase<br>Glutathione peroxidase 1
Cellular response to starvation<br>+<br>Response of EIF2AK4 (GCN2) to amino acid deficiency	14	60S ribosomal protein L18<br>40S ribosomal protein S12<br>60S ribosomal protein L10a<br>60S ribosomal protein L23<br>60S ribosomal protein L4<br>60S ribosomal protein L35<br>60S ribosomal protein L14<br>40S ribosomal protein S8<br>40S ribosomal protein S14

		40S ribosomal protein S10<br>Eukaryotic translation initiation factor 2 subunit 2<br>40S ribosomal protein S24<br>Eukaryotic translation initiation factor 2 subunit 1<br>40S ribosomal protein S6
Unfolded Protein Response (UPR)	7	Endoplasmic reticulum chaperone BiP<br>Protein disulfide-isomerase A6<br>Endoplasmin<br>Eukaryotic translation initiation factor 2 subunit 2<br>Eukaryotic translation initiation factor 2 subunit 1<br>Glutamine--fructose-6-phosphate aminotransferase [isomerizing] 1<br>Talin-1
Immune System	82*	Signal transducer and activator of transcription 2<br>Ubiquitin carboxyl-terminal hydrolase 14<br>Cytoplasmic dynein 1 heavy chain 1<br>Major vault protein<br>Probable E3 ubiquitin-protein ligase HERC4<br>Glycogen phosphorylase, liver form<br>HLA class I histocompatibility antigen, A alpha chain<br>Double-stranded RNA-specific adenosine deaminase<br>Solute carrier family 2, facilitated glucose transporter member 3<br>Thioredoxin<br>26S proteasome non-ATPase regulatory subunit 7<br>Myosin-9<br>Signal transducer and activator of transcription 1-alpha/beta<br>26S proteasome regulatory subunit 8<br>Annexin A1<br>Catalase<br>Actin-related protein 2/3 complex subunit 5<br>Interferon-induced GTP-binding protein Mx2<br>Ras GTPase-activating-like protein IQGAP1<br>Alpha-2-HS-glycoprotein<br>MAP kinase-activated protein kinase 2<br>Cyclin-dependent kinase 11B<br>Annexin A2<br>Protein disulfide-isomerase<br>Signal transducer and activator of transcription 3<br>Stomatin<br>Vitronectin<br>Elongation factor 2<br>26S proteasome non-ATPase regulatory subunit 11<br>Cytoplasmic dynein 1 intermediate chain 2<br>Plastin-2<br>Vinculin<br>Rho-related GTP-binding protein RhoG<br>Tubulin beta-6 chain<br>Endoplasmic reticulum chaperone BiP<br>Synaptosomal-associated protein 23<br>Elongation factor 1-alpha 1<br>Fibrinogen gamma chain<br>U2 small nuclear ribonucleoprotein A'<br>Early endosome antigen 1<br>Actin-related protein 3<br>Gelsolin<br>Proteasome subunit alpha type-6<br>Serine/threonine-protein kinase PAK 2
		Fibrinogen beta chain [Cleaved into: Fibrinopeptide B<br>Fibronectin<br>Glycogenin-1<br>Filamin-A<br>Ferritin heavy chain<br>Rab GDP dissociation inhibitor beta<br>Ferritin light chain<br>Ras GTPase-activating-like protein IQGAP2<br>Proteasome subunit alpha type-5<br>Actin-related protein 2/3 complex subunit 1B<br>Filamin-B<br>Proteasome activator complex subunit 1<br>Protein disulfide-isomerase A3<br>Leukocyte elastase inhibitor<br>26S proteasome regulatory subunit 10B<br>3-ketoacyl-CoA thiolase, peroxisomal<br>Cofilin-1<br>Hemoglobin subunit beta<br>Copine-1<br>Proteasome subunit alpha type-4<br>Moesin<br>Poly<br>Proteasome activator complex subunit 2<br>Cathepsin D<br>Interferon-induced GTP-binding protein Mx1<br>Endoplasmin<br>Catenin beta-1<br>Cytoplasmic FMR1-interacting protein 1<br>Galectin-3<br>Carbonic anhydrase 1<br>F-actin-capping protein subunit alpha-1<br>Vimentin<br>Transcription factor p65<br>Alpha-1-antitrypsin<br>Calpain-1 catalytic subunit<br>Beta-glucuronidase<br>Eukaryotic initiation factor 4A-I<br>Clusterin
Innate Immune System	58	Ubiquitin carboxyl-terminal hydrolase 14<br>Cytoplasmic dynein 1 heavy chain 1<br>Major vault protein<br>Glycogen phosphorylase, liver form<br>Solute carrier family 2, facilitated glucose transporter member 3<br>Thioredoxin<br>26S proteasome non-ATPase regulatory subunit 7<br>Myosin-9<br>26S proteasome regulatory subunit 8<br>Catalase<br>Actin-related protein 2/3 complex subunit 5<br>Ras GTPase-activating-like protein IQGAP1<br>Alpha-2-HS-glycoprotein<br>MAP kinase-activated protein kinase 2<br>Annexin A2<br>Stomatin<br>Vitronectin<br>Elongation factor 2<br>26S proteasome non-ATPase regulatory subunit 11

		Vinculin<br>Rho-related GTP-binding protein RhoG<br>Synaptosomal-associated protein 23<br>Elongation factor 1-alpha 1<br>Fibrinogen gamma chain<br>Early endosome antigen 1<br>Actin-related protein 3<br>Gelsolin<br>Proteasome subunit alpha type-6<br>Serine/threonine-protein kinase PAK 2<br>Fibrinogen beta chain [Cleaved into: Fibrinopeptide B<br>Glycogenin-1<br>Ferritin heavy chain<br>Rab GDP dissociation inhibitor beta<br>Ferritin light chain<br>Ras GTPase-activating-like protein IQGAP2<br>Proteasome subunit alpha type-5<br>Actin-related protein 2/3 complex subunit 1B<br>Proteasome activator complex subunit 1<br>Leukocyte elastase inhibitor<br>26S proteasome regulatory subunit 10B<br>3-ketoacyl-CoA thiolase, peroxisomal<br>Cofilin-1<br>Hemoglobin subunit beta<br>Copine-1<br>Proteasome subunit alpha type-4<br>Poly<br>Proteasome activator complex subunit 2<br>Cathepsin D<br>Endoplasmin<br>Catenin beta-1<br>Cytoplasmic FMR1-interacting protein 1<br>Galectin-3<br>F-actin-capping protein subunit alpha-1<br>Transcription factor p65<br>Alpha-1-antitrypsin<br>Calpain-1 catalytic subunit<br>Beta-glucuronidase<br>Clusterin
Neutrophil degranulation	34	Cytoplasmic dynein 1 heavy chain 1<br>Rab GDP dissociation inhibitor beta<br>Ferritin light chain<br>Major vault protein<br>Ras GTPase-activating-like protein IQGAP2<br>Glycogen phosphorylase, liver form<br>Proteasome subunit alpha type-5<br>Solute carrier family 2, facilitated glucose transporter member 3<br>26S proteasome non-ATPase regulatory subunit 7<br>Leukocyte elastase inhibitor (LEI)<br>3-ketoacyl-CoA thiolase, peroxisomal<br>Hemoglobin subunit beta<br>Actin-related protein 2/3 complex subunit 5<br>Catalase<br>Copine-1<br>Ras GTPase-activating-like protein IQGAP1<br>Alpha-2-HS-glycoprotein<br>Annexin A2

		Stomatin<br>Cathepsin D<br>Elongation factor 2<br>26S proteasome non-ATPase regulatory subunit 11<br>Cytoplasmic FMR1-interacting protein 1<br>Rho-related GTP-binding protein RhoG<br>Galectin-3<br>Vinculin<br>Synaptosomal-associated protein 23<br>Elongation factor 1-alpha 1<br>Alpha-1-antitrypsin<br>Calpain-1 catalytic subunit<br>Gelsolin<br>Beta-glucuronidase<br>Ferritin heavy chain<br>Glycogenin-1
Cytokine Signaling in Immune system	35*	Ubiquitin carboxyl-terminal hydrolase 14<br>Signal transducer and activator of transcription 2<br>Proteasome subunit alpha type-5<br>Double-stranded RNA-specific adenosine deaminase<br>HLA class I histocompatibility antigen, A alpha chain<br>Filamin-B<br>Proteasome activator complex subunit 1<br>26S proteasome non-ATPase regulatory subunit 7<br>Signal transducer and activator of transcription 1-alpha/beta<br>26S proteasome regulatory subunit 10B<br>26S proteasome regulatory subunit 8<br>Cofilin-1<br>Annexin A1<br>Proteasome subunit alpha type-4<br>Interferon-induced GTP-binding protein Mx2<br>MAP kinase-activated protein kinase 2<br>Moesin<br>Annexin A2<br>Protein disulfide-isomerase<br>Signal transducer and activator of transcription 3<br>Proteasome activator complex subunit 2<br>Interferon-induced GTP-binding protein Mx1<br>Endoplasmin<br>26S proteasome non-ATPase regulatory subunit 11<br>Plastin-2<br>Carbonic anhydrase 1<br>F-actin-capping protein subunit alpha-1<br>Vimentin<br>Transcription factor p65<br>U2 small nuclear ribonucleoprotein A'<br>Serine/threonine-protein kinase PAK 2<br>Proteasome subunit alpha type-6<br>Fibronectin<br>Eukaryotic initiation factor 4A-I<br>Filamin-A
Signaling by Interleukins	29**	Ubiquitin carboxyl-terminal hydrolase 14<br>Signal transducer and activator of transcription 2<br>Proteasome subunit alpha type-5<br>Proteasome activator complex subunit 1<br>26S proteasome non-ATPase regulatory subunit 7<br>Signal transducer and activator of transcription 1-alpha/beta

		26S proteasome regulatory subunit 10B<br>26S proteasome regulatory subunit 8<br>Cofilin-1<br>Annexin A1<br>Proteasome subunit alpha type-4<br>MAP kinase-activated protein kinase 2<br>Moesin<br>Annexin A2<br>Protein disulfide-isomerase<br>Signal transducer and activator of transcription 3<br>Proteasome activator complex subunit 2<br>Endoplasmin<br>26S proteasome non-ATPase regulatory subunit 11<br>Plastin-2<br>Carbonic anhydrase 1<br>F-actin-capping protein subunit alpha-1<br>Vimentin<br>Transcription factor p65<br>U2 small nuclear ribonucleoprotein A'<br>Serine/threonine-protein kinase PAK 2<br>Proteasome subunit alpha type-6<br>Fibronectin
Interleukin-1 family signaling	12	Ubiquitin carboxyl-terminal hydrolase 14<br>Proteasome activator complex subunit 2<br>26S proteasome regulatory subunit 10B<br>Proteasome subunit alpha type-5<br>26S proteasome regulatory subunit 8<br>Transcription factor p65<br>Proteasome subunit alpha type-4<br>26S proteasome non-ATPase regulatory subunit 11<br>Proteasome activator complex subunit 1<br>Proteasome subunit alpha type-6<br>26S proteasome non-ATPase regulatory subunit 7<br>Signal transducer and activator of transcription 3
Interleukin-1 signaling	11	Ubiquitin carboxyl-terminal hydrolase 14<br>Proteasome activator complex subunit 2<br>26S proteasome regulatory subunit 10B<br>Proteasome subunit alpha type-5<br>26S proteasome regulatory subunit 8<br>Transcription factor p65<br>Proteasome subunit alpha type-4<br>26S proteasome non-ATPase regulatory subunit 11<br>Proteasome activator complex subunit 1<br>Proteasome subunit alpha type-6<br>26S proteasome non-ATPase regulatory subunit 7
Interleukin-12 family signaling	12*	Signal transducer and activator of transcription 1-alpha/beta<br>Proteasome activator complex subunit 2<br>Carbonic anhydrase 1<br>F-actin-capping protein subunit alpha-1<br>Cofilin-1<br>U2 small nuclear ribonucleoprotein A'<br>Serine/threonine-protein kinase PAK 2<br>Moesin<br>Plastin-2<br>Annexin A2<br>Protein disulfide-isomerase<br>Signal transducer and activator of transcription 3
Interleukin-12 signaling	10	Proteasome activator complex subunit 2<br>Carbonic anhydrase 1<br>F-actin-capping protein subunit alpha-1<br>Cofilin-1<br>U2 small nuclear ribonucleoprotein A'<br>Serine/threonine-protein kinase PAK 2<br>Plastin-2<br>Moesin<br>Annexin A2<br>Protein disulfide-isomerase
Hemostasis	29	Annexin A5<br>Calumenin<br>Guanine nucleotide-binding protein G<br>Melanoma-associated antigen D2<br>Plasminogen<br>Hemoglobin subunit beta<br>Cofilin-1<br>CYFIP-related Rac1 interactor B<br>Apolipoprotein A-I<br>Alpha-2-HS-glycoprotein<br>Serotransferrin<br>Annexin A2<br>Talin-1<br>Transgelin-2<br>Rho-related GTP-binding protein RhoG<br>Vinculin<br>Tubulin beta-6 chain<br>Endoplasmic reticulum chaperone BiP<br>Alpha-actinin-4<br>Kinesin-like protein KIF1B<br>F-actin-capping protein subunit alpha-1<br>Alpha-1-antitrypsin<br>Fibrinogen gamma chain<br>SPARC<br>Guanine nucleotide-binding protein subunit alpha-13<br>Fibrinogen beta chain [Cleaved into: Fibrinopeptide B<br>Fibronectin<br>Clusterin<br>Filamin-A
Platelet activation, signaling and aggregation	24	Annexin A5<br>Calumenin<br>Guanine nucleotide-binding protein G<br>Transgelin-2<br>Melanoma-associated antigen D2<br>Rho-related GTP-binding protein RhoG<br>Vinculin<br>Endoplasmic reticulum chaperone BiP<br>Alpha-actinin-4<br>Plasminogen<br>Cofilin-1<br>CYFIP-related Rac1 interactor B<br>Alpha-1-antitrypsin<br>Apolipoprotein A-I<br>Fibrinogen gamma chain<br>Alpha-2-HS-glycoprotein<br>Serotransferrin<br>SPARC

		Guanine nucleotide-binding protein subunit alpha-13<br>Fibrinogen beta chain [Cleaved into: Fibrinopeptide B<br>Fibronectin<br>Clusterin<br>Talin-1<br>Filamin-A
Platelet Aggregation (Plug Formation)	4	Fibrinogen gamma chain<br>Fibrinogen beta chain<br>Fibronectin<br>Talin-1
Response to elevated platelet cytosolic Ca2+<br>+<br>Platelet degranulation	21	Annexin A5<br>Calumenin<br>Transgelin-2<br>Melanoma-associated antigen D2<br>Vinculin<br>Endoplasmic reticulum chaperone BiP<br>Alpha-actinin-4<br>Plasminogen<br>Cofilin-1<br>CYFIP-related Rac1 interactor B<br>Alpha-1-antitrypsin<br>Apolipoprotein A-I<br>Fibrinogen gamma chain<br>Serotransferrin<br>Alpha-2-HS-glycoprotein<br>SPARC<br>Fibrinogen beta chain [Cleaved into: Fibrinopeptide B<br>Fibronectin<br>Clusterin<br>Talin-1<br>Filamin-A
Nervous system development<br>+<br>Axon guidance	37	40S ribosomal protein S12<br>Proteasome subunit alpha type-5<br>Actin-related protein 2/3 complex subunit 1B<br>Polyadenylate-binding protein 1<br>Proteasome activator complex subunit 1<br>26S proteasome non-ATPase regulatory subunit 7<br>Myosin-9<br>Ezrin<br>Eukaryotic peptide chain release factor subunit 1<br>60S ribosomal protein L35<br>26S proteasome regulatory subunit 10B<br>60S ribosomal protein L14<br>26S proteasome regulatory subunit 8<br>Radixin<br>Cofilin-1<br>Actin-related protein 2/3 complex subunit 5<br>Proteasome subunit alpha type-4<br>40S ribosomal protein S10<br>Moesin<br>40S ribosomal protein S6<br>Talin-1<br>60S ribosomal protein L18<br>Proteasome activator complex subunit 2<br>60S ribosomal protein L10a<br>60S ribosomal protein L23<br>60S ribosomal protein L4

		26S proteasome non-ATPase regulatory subunit 11<br>Myosin regulatory light chain 12A<br>Tubulin beta-6 chain<br>40S ribosomal protein S14<br>40S ribosomal protein S8<br>Actin-related protein 3<br>40S ribosomal protein S24<br>Ran-binding protein 9<br>Serine/threonine-protein kinase PAK 2<br>Proteasome subunit alpha type-6<br>Regulator of nonsense transcripts 2
Signaling by ROBO receptors	25	40S ribosomal protein S12<br>Proteasome subunit alpha type-5<br>Polyadenylate-binding protein 1<br>Proteasome activator complex subunit 1<br>26S proteasome non-ATPase regulatory subunit 7<br>Eukaryotic peptide chain release factor subunit 1<br>60S ribosomal protein L35<br>26S proteasome regulatory subunit 10B<br>60S ribosomal protein L14<br>26S proteasome regulatory subunit 8<br>Proteasome subunit alpha type-4<br>40S ribosomal protein S10<br>40S ribosomal protein S6<br>60S ribosomal protein L18<br>Proteasome activator complex subunit 2<br>60S ribosomal protein L10a<br>60S ribosomal protein L23<br>60S ribosomal protein L4<br>26S proteasome non-ATPase regulatory subunit 11<br>40S ribosomal protein S14<br>40S ribosomal protein S8<br>40S ribosomal protein S24<br>Serine/threonine-protein kinase PAK 2<br>Proteasome subunit alpha type-6<br>Regulator of nonsense transcripts 2
Regulation of expression of SLITs and ROBOs	24	60S ribosomal protein L18<br>Proteasome activator complex subunit 2<br>40S ribosomal protein S12<br>Proteasome subunit alpha type-5<br>60S ribosomal protein L10a<br>60S ribosomal protein L23<br>60S ribosomal protein L4<br>Polyadenylate-binding protein 1<br>26S proteasome non-ATPase regulatory subunit 11<br>Proteasome activator complex subunit 1<br>26S proteasome non-ATPase regulatory subunit 7<br>Eukaryotic peptide chain release factor subunit 1<br>60S ribosomal protein L35<br>26S proteasome regulatory subunit 10B<br>60S ribosomal protein L14<br>26S proteasome regulatory subunit 8<br>40S ribosomal protein S14<br>40S ribosomal protein S8<br>Proteasome subunit alpha type-4<br>40S ribosomal protein S10<br>40S ribosomal protein S24

		Proteasome subunit alpha type-6<br>Regulator of nonsense transcripts 2<br>40S ribosomal protein S6
Metabolism of proteins	80	Mothers against decapentaplegic homolog 4<br>Ubiquitin carboxyl-terminal hydrolase 14<br>Cytoplasmic dynein 1 heavy chain 1<br>Golgi-specific brefeldin A-resistance guanine nucleotide exchange factor 1<br>HLA class I histocompatibility antigen, A alpha chain<br>ADP-ribosylation factor 4<br>Thioredoxin<br>Spondin-1<br>26S proteasome non-ATPase regulatory subunit 7<br>E3 ubiquitin-protein ligase BRE1B<br>Plasminogen<br>26S proteasome regulatory subunit 8<br>Apolipoprotein E<br>Apolipoprotein A-I<br>40S ribosomal protein S10<br>Alpha-2-HS-glycoprotein<br>40S ribosomal protein S6<br>Protein disulfide-isomerase<br>Signal transducer and activator of transcription 3<br>60S ribosomal protein L18<br>Coatomer subunit beta'<br>Elongation factor 2<br>26S proteasome non-ATPase regulatory subunit 11<br>Cadherin-2<br>60S ribosomal protein L4<br>Cytoplasmic dynein 1 intermediate chain 2<br>Glutamine--fructose-6-phosphate aminotransferase [isomerizing] 1<br>Tubulin beta-6 chain<br>Elongation factor 1-alpha 1<br>40S ribosomal protein S8<br>Fibrinogen gamma chain<br>Eukaryotic translation initiation factor 2 subunit 2<br>40S ribosomal protein S24<br>Gelsolin<br>Proteasome subunit alpha type-6<br>Eukaryotic translation initiation factor 3 subunit E<br>Fibronectin<br>Coatomer subunit alpha<br>Ceruloplasmin<br>40S ribosomal protein S12<br>Proteasome subunit alpha type-5<br>Calumenin<br>Sec1 family domain-containing protein 1<br>Polyadenylate-binding protein 1<br>Proteasome activator complex subunit 1<br>Asparagine--tRNA ligase, cytoplasmic<br>Osteopontin<br>Protein disulfide-isomerase A3<br>Eukaryotic peptide chain release factor subunit 1<br>60S ribosomal protein L35<br>Probable ATP-dependent RNA helicase DDX5<br>Ubiquitin carboxyl-terminal hydrolase 15
		26S proteasome regulatory subunit 10B<br>60S ribosomal protein L14<br>LIM and cysteine-rich domains protein 1<br>Protein disulfide-isomerase A6<br>Proteasome subunit alpha type-4<br>Eukaryotic translation initiation factor 5<br>DNA damage-binding protein 1<br>Eukaryotic translation initiation factor 3 subunit A<br>Eukaryotic translation initiation factor 2 subunit 1<br>Serotransferrin<br>Moesin<br>Proteasome activator complex subunit 2<br>Cathepsin D<br>Translocon-associated protein subunit delta<br>60S ribosomal protein L23<br>60S ribosomal protein L10a<br>Tryptophan--tRNA ligase, cytoplasmic<br>Endoplasmin<br>Catenin beta-1<br>Proliferating cell nuclear antigen<br>Mannose-1-phosphate guanyltransferase beta<br>F-actin-capping protein subunit alpha-1<br>Transcription factor p65<br>40S ribosomal protein S14<br>Alpha-1-antitrypsin<br>Tenascin<br>Eukaryotic translation initiation factor 3 subunit L<br>Eukaryotic initiation factor 4A-I
Translation	26	40S ribosomal protein S12<br>Polyadenylate-binding protein 1<br>Asparagine--tRNA ligase, cytoplasmic<br>Eukaryotic peptide chain release factor subunit 1<br>60S ribosomal protein L35<br>60S ribosomal protein L14<br>Eukaryotic translation initiation factor 5<br>40S ribosomal protein S10<br>Eukaryotic translation initiation factor 3 subunit A<br>Eukaryotic translation initiation factor 2 subunit 1<br>40S ribosomal protein S6<br>60S ribosomal protein L18<br>Elongation factor 2<br>Translocon-associated protein subunit delta<br>60S ribosomal protein L10a<br>Tryptophan--tRNA ligase, cytoplasmic<br>60S ribosomal protein L23<br>60S ribosomal protein L4<br>Elongation factor 1-alpha 1<br>40S ribosomal protein S14<br>40S ribosomal protein S8<br>Eukaryotic translation initiation factor 3 subunit L<br>Eukaryotic translation initiation factor 2 subunit 2<br>40S ribosomal protein S24<br>Eukaryotic translation initiation factor 3 subunit E<br>Eukaryotic initiation factor 4A-I
Post-translational	15	Ceruloplasmin<br>Calumenin<br>Endoplasmin

protein phosphorylation		Cadherin-2<br>Osteopontin<br>Protein disulfide-isomerase A6<br>Apolipoprotein E<br>Alpha-1-antitrypsin<br>Apolipoprotein A-I<br>Fibrinogen gamma chain<br>Tenascin<br>Serotransferrin<br>Alpha-2-HS-glycoprotein<br>Protein disulfide-isomerase<br>Fibronectin
Regulation of Insulin-like Growth Factor (IGF) transport and uptake by Insulin-like Growth Factor Binding Proteins (IGFBPs)	16	Ceruloplasmin<br>Calumenin<br>Endoplasmin<br>Cadherin-2<br>Osteopontin<br>Plasminogen<br>Protein disulfide-isomerase A6<br>Apolipoprotein E<br>Alpha-1-antitrypsin<br>Apolipoprotein A-I<br>Fibrinogen gamma chain<br>Tenascin<br>Serotransferrin<br>Alpha-2-HS-glycoprotein<br>Protein disulfide-isomerase<br>Fibronectin
Metabolism of RNA	34	40S ribosomal protein S12<br>Proteasome subunit alpha type-5<br>Double-stranded RNA-specific adenosine deaminase<br>THO complex subunit 4<br>Polyadenylate-binding protein 1<br>Proteasome activator complex subunit 1<br>26S proteasome non-ATPase regulatory subunit 7<br>Insulin-like growth factor 2 mRNA-binding protein 2<br>Eukaryotic peptide chain release factor subunit 1<br>60S ribosomal protein L35<br>Probable ATP-dependent RNA helicase DDX5<br>26S proteasome regulatory subunit 10B<br>60S ribosomal protein L14<br>26S proteasome regulatory subunit 8<br>Proteasome subunit alpha type-4<br>40S ribosomal protein S10<br>MAP kinase-activated protein kinase 2<br>Pre-mRNA 3'-end-processing factor FIP1<br>Polypyrimidine tract-binding protein 1<br>Poly<br>40S ribosomal protein S6<br>60S ribosomal protein L18<br>Proteasome activator complex subunit 2<br>60S ribosomal protein L10a<br>60S ribosomal protein L23<br>60S ribosomal protein L4<br>26S proteasome non-ATPase regulatory subunit 11<br>40S ribosomal protein S14<br>40S ribosomal protein S8

		U2 small nuclear ribonucleoprotein A'<br>40S ribosomal protein S24<br>Proteasome subunit alpha type-6<br>Regulator of nonsense transcripts 2<br>Eukaryotic initiation factor 4A-I
Programmed Cell Death	17	Plectin<br>Proteasome activator complex subunit 2<br>Flotillin-1<br>Proteasome subunit alpha type-5<br>26S proteasome non-ATPase regulatory subunit 11<br>Proteasome activator complex subunit 1<br>Catenin beta-1<br>26S proteasome non-ATPase regulatory subunit 7<br>26S proteasome regulatory subunit 10B<br>26S proteasome regulatory subunit 8<br>Vimentin<br>Proteasome subunit alpha type-4<br>Gelsolin<br>Serine/threonine-protein kinase PAK 2<br>Proteasome subunit alpha type-6<br>Flotillin-2<br>Signal transducer and activator of transcription 3
Apoptosis	15	Plectin<br>Proteasome activator complex subunit 2<br>Proteasome subunit alpha type-5<br>26S proteasome non-ATPase regulatory subunit 11<br>Proteasome activator complex subunit 1<br>Catenin beta-1<br>26S proteasome non-ATPase regulatory subunit 7<br>26S proteasome regulatory subunit 10B<br>26S proteasome regulatory subunit 8<br>Vimentin<br>Proteasome subunit alpha type-4<br>Gelsolin<br>Serine/threonine-protein kinase PAK 2<br>Proteasome subunit alpha type-6<br>Signal transducer and activator of transcription 3
Regulation of Apoptosis	10	Proteasome activator complex subunit 2<br>26S proteasome regulatory subunit 10B<br>Proteasome subunit alpha type-5<br>26S proteasome regulatory subunit 8<br>Proteasome subunit alpha type-4<br>26S proteasome non-ATPase regulatory subunit 11<br>Proteasome activator complex subunit 1<br>Serine/threonine-protein kinase PAK 2<br>Proteasome subunit alpha type-6<br>26S proteasome non-ATPase regulatory subunit 7

***:** Signal transducer and activator of transcription 1-alpha/beta was counted as 3 entities in Reactome
****:** Signal transducer and activator of transcription 1-alpha/beta was counted as 2 entities in Reactome

Selected downregulated pathways		
**Pathway name**	**Number of proteins**	**Protein names**
Nervous system development<br>+<br>Axon guidance	29	Dynamin-1<br>Spectrin beta chain, non-erythrocytic 2<br>Contactin-1<br>Spectrin alpha chain, non-erythrocytic 1<br>CLIP-associating protein 2<br>Dynamin-3<br>Neural cell adhesion molecule L1<br>Endophilin-A1<br>Neural cell adhesion molecule 1<br>Protein kinase C alpha type<br>Clathrin heavy chain 1<br>cAMP-dependent protein kinase catalytic subunit beta<br>Dihydropyrimidinase-related protein 1<br>Clathrin light chain B<br>Dihydropyrimidinase-related protein 2<br>Rap1 GTPase-activating protein 1<br>Contactin-associated protein 1<br>Neurofascin<br>Ankyrin-3<br>Tubulin beta-3 chain<br>Ankyrin-2<br>Spectrin beta chain, non-erythrocytic 1<br>Dihydropyrimidinase-related protein 5<br>AP-2 complex subunit alpha-1<br>cAMP-dependent protein kinase catalytic subunit alpha<br>Tubulin alpha-4A chain<br>Dihydropyrimidinase-related protein 4<br>Tubulin beta-4A chain<br>Neuronal cell adhesion molecule
L1CAM interactions	20	Contactin-associated protein 1<br>Dynamin-1<br>Spectrin beta chain, non-erythrocytic 2<br>Contactin-1<br>Spectrin alpha chain, non-erythrocytic 1<br>Neurofascin<br>Ankyrin-3<br>Tubulin beta-3 chain<br>Dynamin-3<br>Ankyrin-2<br>Spectrin beta chain, non-erythrocytic 1<br>Neural cell adhesion molecule L1<br>AP-2 complex subunit alpha-1<br>Endophilin-A1<br>Neural cell adhesion molecule 1<br>Clathrin heavy chain 1<br>Tubulin alpha-4A chain<br>Tubulin beta-4A chain<br>Dihydropyrimidinase-related protein 2<br>Neuronal cell adhesion molecule
Metabolism	82	Potassium voltage-gated channel subfamily B member 1<br>Electrogenic aspartate/glutamate antiporter SLC25A12, mitochondrial<br>Glutathione hydrolase 7<br>Pyruvate dehydrogenase E1 component subunit alpha, somatic form, mitochondrial<br>Malate dehydrogenase, mitochondrial<br>Dihydrolipoyllysine-residue acetyltransferase component of pyruvate dehydrogenase complex, mitochondrial<br>Malate dehydrogenase, cytoplasmic<br>NADH dehydrogenase [ubiquinone] 1 alpha subcomplex subunit 2<br>ATP synthase subunit gamma, mitochondrial<br>Iron-sulfur cluster assembly 2 homolog, mitochondrial<br>Isocitrate dehydrogenase [NAD] subunit alpha, mitochondrial<br>5'-3' exonuclease PLD3<br>Isocitrate dehydrogenase [NAD] subunit beta, mitochondrial<br>Creatine kinase U-type, mitochondrial<br>Succinate--CoA ligase [ADP-forming] subunit beta, mitochondrial<br>NADH dehydrogenase [ubiquinone] flavoprotein 2, mitochondrial<br>Citrate synthase, mitochondrial<br>Mitochondrial 2-oxoglutarate/malate carrier protein<br>Aspartate aminotransferase, mitochondrial<br>Leucine-rich PPR motif-containing protein, mitochondrial<br>L-lactate dehydrogenase B chain<br>Triosephosphate isomerase<br>Adenylosuccinate synthetase isozyme 1<br>Fructose-bisphosphate aldolase C<br>NADH dehydrogenase [ubiquinone] 1 alpha subcomplex subunit 9, mitochondrial<br>Electrogenic aspartate/glutamate antiporter SLC25A13, mitochondrial<br>cAMP-dependent protein kinase catalytic subunit beta<br>Guanine nucleotide-binding protein G<br>Cytochrome c oxidase subunit 5A, mitochondrial<br>Fumarate hydratase, mitochondrial<br>Cytochrome c oxidase subunit 5B, mitochondrial<br>Isocitrate dehydrogenase [NAD] subunit gamma, mitochondrial<br>Retinol dehydrogenase 11<br>NADH-ubiquinone oxidoreductase 75 kDa subunit, mitochondrial<br>ATP synthase subunit O, mitochondrial<br>Cytosolic acyl coenzyme A thioester hydrolase<br>Prostaglandin E synthase 2<br>Pyruvate dehydrogenase E1 component subunit beta, mitochondrial<br>Mitochondrial pyruvate carrier 2<br>NADH dehydrogenase [ubiquinone] iron-sulfur protein 7, mitochondrial<br>Guanine nucleotide-binding protein G<br>Aconitate hydratase, mitochondrial<br>Guanine nucleotide-binding protein G<br>AMP deaminase 2<br>Cytochrome c1, heme protein, mitochondrial<br>Gamma-enolase<br>Dihydrolipoyl dehydrogenase, mitochondrial<br>Guanine nucleotide-binding protein subunit alpha-11<br>ATP synthase subunit f, mitochondrial<br>Vesicle-associated membrane protein-associated protein B/C

		Membrane-associated phosphatidylinositol transfer protein 1<br>Guanine nucleotide-binding protein G<br>ATP synthase protein 8<br>Glutamine synthetase<br>Guanine nucleotide-binding protein G<br>ATP synthase subunit alpha, mitochondrial<br>Adenosylhomocysteinase<br>ATP synthase-coupling factor 6, mitochondrial<br>ATP synthase F<br>Protein kinase C alpha type<br>Aspartate aminotransferase, cytoplasmic<br>Succinate dehydrogenase [ubiquinone] flavoprotein subunit, mitochondrial<br>ATP synthase subunit e, mitochondrial<br>Glutaminase kidney isoform, mitochondrial<br>Adenylate kinase isoenzyme 1<br>Cytochrome c oxidase subunit 2<br>L-2-hydroxyglutarate dehydrogenase, mitochondrial<br>Vesicle-associated membrane protein 2<br>Dihydrolipoyllysine-residue succinyltransferase component of 2-oxoglutarate dehydrogenase complex, mitochondrial<br>Succinate--CoA ligase [ADP/GDP-forming] subunit alpha, mitochondrial<br>Gephyrin [Includes: Molybdopterin adenylyltransferase<br>Oxysterol-binding protein-related protein 1<br>Phosphatidylinositol 5-phosphate 4-kinase type-2 beta<br>Synaptojanin-1<br>Methylglutaconyl-CoA hydratase, mitochondrial<br>Hexokinase-1<br>cAMP-dependent protein kinase catalytic subunit alpha<br>Synaptosomal-associated protein 25<br>NADH dehydrogenase [ubiquinone] flavoprotein 1, mitochondrial<br>Guanine nucleotide-binding protein subunit beta-5<br>Voltage-dependent anion-selective channel protein 1<br>ATP synthase subunit beta, mitochondrial
Integration of energy metabolism	13	Potassium voltage-gated channel subfamily B member 1<br>Guanine nucleotide-binding protein subunit alpha-11<br>Guanine nucleotide-binding protein G<br>Guanine nucleotide-binding protein G<br>cAMP-dependent protein kinase catalytic subunit alpha<br>Guanine nucleotide-binding protein G<br>Guanine nucleotide-binding protein G<br>Synaptosomal-associated protein 25<br>Protein kinase C alpha type<br>cAMP-dependent protein kinase catalytic subunit beta<br>Guanine nucleotide-binding protein subunit beta-5<br>Guanine nucleotide-binding protein G<br>Vesicle-associated membrane protein 2
The citric acid (TCA) cycle and respiratory electron transport	39	Pyruvate dehydrogenase E1 component subunit alpha, somatic form, mitochondrial<br>Malate dehydrogenase, mitochondrial<br>Dihydrolipoyllysine-residue acetyltransferase component of pyruvate dehydrogenase complex, mitochondrial<br>ATP synthase subunit f, mitochondrial<br>NADH dehydrogenase [ubiquinone] 1 alpha subcomplex subunit 2<br>ATP synthase subunit gamma, mitochondrial
		Isocitrate dehydrogenase [NAD] subunit alpha, mitochondrial<br>ATP synthase protein 8<br>Isocitrate dehydrogenase [NAD] subunit beta, mitochondrial<br>Succinate--CoA ligase [ADP-forming] subunit beta, mitochondrial<br>NADH dehydrogenase [ubiquinone] flavoprotein 2, mitochondrial<br>Citrate synthase, mitochondrial<br>ATP synthase subunit alpha, mitochondrial<br>Leucine-rich PPR motif-containing protein, mitochondrial<br>ATP synthase-coupling factor 6, mitochondrial<br>ATP synthase F<br>Succinate dehydrogenase [ubiquinone] flavoprotein subunit, mitochondrial<br>L-lactate dehydrogenase B chain<br>ATP synthase subunit e, mitochondrial<br>Cytochrome c oxidase subunit 2<br>NADH dehydrogenase [ubiquinone] 1 alpha subcomplex subunit 9, mitochondrial<br>L-2-hydroxyglutarate dehydrogenase, mitochondrial<br>Cytochrome c oxidase subunit 5A, mitochondrial<br>Fumarate hydratase, mitochondrial<br>Cytochrome c oxidase subunit 5B, mitochondrial<br>Isocitrate dehydrogenase [NAD] subunit gamma, mitochondrial<br>Succinate--CoA ligase [ADP/GDP-forming] subunit alpha, mitochondrial<br>Dihydrolipoyllysine-residue succinyltransferase component of 2-oxoglutarate dehydrogenase complex, mitochondrial<br>ATP synthase subunit O, mitochondrial<br>NADH-ubiquinone oxidoreductase 75 kDa subunit, mitochondrial<br>Pyruvate dehydrogenase E1 component subunit beta, mitochondrial<br>Mitochondrial pyruvate carrier 2<br>NADH dehydrogenase [ubiquinone] iron-sulfur protein 7, mitochondrial<br>Aconitate hydratase, mitochondrial<br>Cytochrome c1, heme protein, mitochondrial<br>NADH dehydrogenase [ubiquinone] flavoprotein 1, mitochondrial<br>Voltage-dependent anion-selective channel protein 1<br>ATP synthase subunit beta, mitochondrial<br>Dihydrolipoyl dehydrogenase, mitochondrial
Neuronal System	42	Band 4.1-like protein 1<br>Calcium/calmodulin-dependent protein kinase type II subunit gamma<br>Potassium voltage-gated channel subfamily B member 1<br>Calcium/calmodulin-dependent protein kinase type II subunit beta<br>Guanine nucleotide-binding protein G<br>Reticulon-3<br>Glutamine synthetase<br>Synapsin-1<br>Guanine nucleotide-binding protein G<br>Glutamate decarboxylase 2<br>Ras-related protein Rab-3A<br>Neurexin-3<br>Neurofilament light polypeptide<br>Protein kinase C alpha type<br>Glutaminase kidney isoform, mitochondrial<br>cAMP-dependent protein kinase catalytic subunit beta

		Vesicle-associated membrane protein 2<br>Guanine nucleotide-binding protein G<br>Synapsin-2<br>4-aminobutyrate aminotransferase, mitochondrial<br>Disks large homolog 2<br>Gamma-aminobutyric acid receptor subunit beta-2<br>Gamma-aminobutyric acid receptor subunit beta-3<br>Tubulin beta-3 chain<br>Vesicular inhibitory amino acid transporter<br>Neuroligin-2<br>Vesicular glutamate transporter 1<br>Band 4.1-like protein 3<br>AP-2 complex subunit alpha-1<br>cAMP-dependent protein kinase catalytic subunit alpha<br>Guanine nucleotide-binding protein G<br>Glutamate decarboxylase 1<br>Synaptotagmin-1<br>Peripheral plasma membrane protein CASK<br>Guanine nucleotide-binding protein G<br>Synaptosomal-associated protein 25<br>Tubulin alpha-4A chain<br>Tubulin beta-4A chain<br>Guanine nucleotide-binding protein subunit beta-5<br>Complexin-1<br>Protein phosphatase 1E<br>Vesicle-fusing ATPase
Transmission across Chemical Synapses	37	Band 4.1-like protein 1<br>Calcium/calmodulin-dependent protein kinase type II subunit gamma<br>Calcium/calmodulin-dependent protein kinase type II subunit beta<br>Guanine nucleotide-binding protein G<br>Glutamine synthetase<br>Synapsin-1<br>Glutamate decarboxylase 2<br>Guanine nucleotide-binding protein G<br>Ras-related protein Rab-3A<br>Neurofilament light polypeptide<br>Protein kinase C alpha type<br>Glutaminase kidney isoform, mitochondrial<br>cAMP-dependent protein kinase catalytic subunit beta<br>Guanine nucleotide-binding protein G<br>Vesicle-associated membrane protein 2<br>Synapsin-2<br>4-aminobutyrate aminotransferase, mitochondrial<br>Disks large homolog 2<br>Gamma-aminobutyric acid receptor subunit beta-2<br>Gamma-aminobutyric acid receptor subunit beta-3<br>Tubulin beta-3 chain<br>Vesicular inhibitory amino acid transporter<br>Vesicular glutamate transporter 1<br>AP-2 complex subunit alpha-1<br>cAMP-dependent protein kinase catalytic subunit alpha<br>Guanine nucleotide-binding protein G<br>Peripheral plasma membrane protein CASK<br>Glutamate decarboxylase 1<br>Synaptotagmin-1
		Guanine nucleotide-binding protein G<br>Synaptosomal-associated protein 25<br>Tubulin alpha-4A chain<br>Tubulin beta-4A chain<br>Guanine nucleotide-binding protein subunit beta-5<br>Complexin-1<br>Protein phosphatase 1E<br>Vesicle-fusing ATPase
Neurotransmitter release cycle	14	Vesicular inhibitory amino acid transporter<br>Vesicular glutamate transporter 1<br>Synapsin-1<br>Glutamate decarboxylase 2<br>Ras-related protein Rab-3A<br>Glutamate decarboxylase 1<br>Peripheral plasma membrane protein CASK<br>Synaptotagmin-1<br>Synaptosomal-associated protein 25<br>Glutaminase kidney isoform, mitochondrial<br>Vesicle-associated membrane protein 2<br>Complexin-1<br>Synapsin-2<br>4-aminobutyrate aminotransferase, mitochondrial
Neurotransmitter receptors and postsynaptic signal transmission	23	Band 4.1-like protein 1<br>Calcium/calmodulin-dependent protein kinase type II subunit gamma<br>Disks large homolog 2<br>Calcium/calmodulin-dependent protein kinase type II subunit beta<br>Gamma-aminobutyric acid receptor subunit beta-2<br>Gamma-aminobutyric acid receptor subunit beta-3<br>Tubulin beta-3 chain<br>Guanine nucleotide-binding protein G<br>AP-2 complex subunit alpha-1<br>cAMP-dependent protein kinase catalytic subunit alpha<br>Guanine nucleotide-binding protein G<br>Guanine nucleotide-binding protein G<br>Peripheral plasma membrane protein CASK<br>Guanine nucleotide-binding protein G<br>Neurofilament light polypeptide<br>Protein kinase C alpha type<br>Tubulin alpha-4A chain<br>Tubulin beta-4A chain<br>cAMP-dependent protein kinase catalytic subunit beta<br>Guanine nucleotide-binding protein subunit beta-5<br>Guanine nucleotide-binding protein G<br>Protein phosphatase 1E<br>Vesicle-fusing ATPase
Organelle biogenesis and maintenance	20	ATP synthase subunit O, mitochondrial<br>ATP synthase subunit f, mitochondrial<br>Tubulin beta-3 chain<br>ATP synthase subunit gamma, mitochondrial<br>ATP synthase protein 8<br>Stress-70 protein, mitochondrial<br>cAMP-dependent protein kinase catalytic subunit alpha<br>14-3-3 protein gamma<br>ATP synthase subunit alpha, mitochondrial<br>ATP synthase-coupling factor 6, mitochondrial

		ATP synthase F<br>Dynein light chain 2, cytoplasmic<br>Tubulin alpha-4A chain<br>Rab-3A-interacting protein<br>ATP synthase subunit e, mitochondrial<br>Tubulin beta-4A chain<br>Platelet-activating factor acetylhydrolase IB subunit beta<br>MICOS complex subunit MIC60<br>ATP synthase subunit beta, mitochondrial<br>Superoxide dismutase [Mn], mitochondrial
Mitochondrial biogenesis	12	ATP synthase subunit alpha, mitochondrial<br>ATP synthase-coupling factor 6, mitochondrial<br>ATP synthase subunit O, mitochondrial<br>ATP synthase F<br>ATP synthase subunit f, mitochondrial<br>ATP synthase subunit e, mitochondrial<br>ATP synthase subunit gamma, mitochondrial<br>ATP synthase protein 8<br>MICOS complex subunit MIC60<br>ATP synthase subunit beta, mitochondrial<br>Stress-70 protein, mitochondrial<br>Superoxide dismutase [Mn], mitochondrial
Cristae formation	11	ATP synthase subunit alpha, mitochondrial<br>ATP synthase-coupling factor 6, mitochondrial<br>ATP synthase subunit O, mitochondrial<br>ATP synthase F<br>ATP synthase subunit f, mitochondrial<br>ATP synthase subunit e, mitochondrial<br>ATP synthase subunit gamma, mitochondrial<br>ATP synthase protein 8<br>MICOS complex subunit MIC60<br>ATP synthase subunit beta, mitochondrial<br>Stress-70 protein, mitochondrial
Protein localization	14	Electrogenic aspartate/glutamate antiporter SLC25A12, mitochondrial<br>Isocitrate dehydrogenase [NAD] subunit gamma, mitochondrial<br>ADP/ATP translocase 1<br>Stress-70 protein, mitochondrial<br>Mitochondrial import receptor subunit TOM22 homolog<br>Citrate synthase, mitochondrial<br>Aconitate hydratase, mitochondrial<br>ATP synthase subunit alpha, mitochondrial<br>Mitochondrial import receptor subunit TOM70<br>Cytochrome c1, heme protein, mitochondrial<br>Electrogenic aspartate/glutamate antiporter SLC25A13, mitochondrial<br>Vesicle-associated membrane protein 2<br>Voltage-dependent anion-selective channel protein 1<br>ATP synthase subunit beta, mitochondrial
Mitochondrial protein import	13	Electrogenic aspartate/glutamate antiporter SLC25A12, mitochondrial<br>Isocitrate dehydrogenase [NAD] subunit gamma, mitochondrial<br>ADP/ATP translocase 1<br>Stress-70 protein, mitochondrial<br>Mitochondrial import receptor subunit TOM22 homolog<br>Citrate synthase, mitochondrial<br>Aconitate hydratase, mitochondrial
		ATP synthase subunit alpha, mitochondrial<br>Mitochondrial import receptor subunit TOM70<br>Cytochrome c1, heme protein, mitochondrial<br>Electrogenic aspartate/glutamate antiporter SLC25A13, mitochondrial<br>Voltage-dependent anion-selective channel protein 1<br>ATP synthase subunit beta, mitochondrial
Visual phototransduction	9	Guanylyl cyclase-activating protein 1<br>cGMP-gated cation channel alpha-1<br>Guanine nucleotide-binding protein G<br>Retinol dehydrogenase 11<br>Protein kinase C alpha type<br>Short-wave-sensitive opsin 1<br>Guanine nucleotide-binding protein G<br>Guanine nucleotide-binding protein subunit beta-5<br>Guanine nucleotide-binding protein G
The phototransduction cascade	7	Guanylyl cyclase-activating protein 1<br>cGMP-gated cation channel alpha-1<br>Guanine nucleotide-binding protein G<br>Protein kinase C alpha type<br>Guanine nucleotide-binding protein G<br>Guanine nucleotide-binding protein subunit beta-5<br>Guanine nucleotide-binding protein G
